# Whole Resistome Analysis in *Campylobacter jejuni* and *C. coli* Genomes Available in Public Repositories

**DOI:** 10.3389/fmicb.2021.662144

**Published:** 2021-07-05

**Authors:** José F. Cobo-Díaz, Paloma González del Río, Avelino Álvarez-Ordóñez

**Affiliations:** ^1^Department of Food Hygiene and Technology, Universidad de León, León, Spain; ^2^Institute of Food Science and Technology, Universidad de León, León, Spain

**Keywords:** *Campylobacter jejuni*, resistome, whole genome sequencing, host specialization, livestock and human sources, *Campylobacter coli*

## Abstract

*Campylobacter* spp. are the most frequent agent of human gastroenteritis worldwide, and the spread of multidrug-resistant strains makes the clinical treatment difficult. The current study presents the resistome analysis of 39,798 *Campylobacter jejuni* and 11,920 *Campylobacter coli* genomes available in public repositories. Determinants of resistance to β-lactams (Be) and tetracyclines (Te) were the most frequent for both species, with resistance to quinolones (Qu) as the third most important on *C. jejuni* and to aminoglycosides (Am) on *C. coli*. Moreover, resistance to Te, Qu, and Am was frequently found in co-occurrence with resistance to other antibiotic families. Geographical differences on clonal complexes distribution were found for *C. jejuni* and on resistome genotypes for both *C. jejuni* and *C. coli* species. Attending to the resistome patterns by isolation source, three main clusters of genomes were found on *C. jejuni* genomes at antimicrobial resistance gene level. The first cluster was formed by genomes from human, food production animals (e.g., sheep, cow, and chicken), and food (e.g., dairy products) isolates. The higher incidence of *tet(O)*, associated with tetracycline resistance, and the *gyrA* (T86I) single-nucleotide polymorphism (SNP), associated with quinolone resistance, among genomes from this cluster could be due to the intense use of these antibiotics in veterinary and human clinical settings. Similarly, a high incidence of *tet(O)* genes of *C. coli* genomes from pig, cow, and turkey was found. Moreover, the cluster based on resistome patterns formed by *C. jejuni* and *C. coli* genomes of human, turkey, and chicken origin is in agreement with previous observations reporting chicken or poultry-related environments as the main source of human campylobacteriosis infections. Most clonal complexes (CCs) associated with chicken host specialization (e.g., ST-354, ST-573, ST-464, and ST-446) were the CCs with the highest prevalence of determinants of resistance to Be, Qu, and Te. Finally, a clear trend toward an increase in the occurrence of Te and Qu resistance determinants on *C. jejuni*, linked to the spread of the co-occurrence of the *bla*_*OXA–*61_ and *tet(O)-tet(O/W/O)* genes and the *gyrA* (T86I) SNP, was found from 2001 to date in Europe.

## Introduction

Thermophilic *Campylobacter* species, and specially *Campylobacter jejuni* and *Campylobacter coli*, are the most frequent agents of human gastroenteritis both in industrialized countries (Kaakoush et al., 2015; European Food Safety Authority, 2018) and in low- and middle-income countries (Fischer Walker et al., 2013; Liu et al., 2015), with *C. jejuni* accounting for up to 80% of all human *Campylobacter* gastroenteritis cases (Bronnec et al., 2016), with estimations of over 800,000 cases per year between 2000 and 2008 (Scallan et al., 2011). Moreover, *C. jejuni* is the most frequent foodborne bacterial pathogen globally, with developing countries being the most affected (Yahara et al., 2017).

*Campylobacter jejuni* and *C. coli* are present in the digestive tract of different animals, including birds, cattle, sheep, pigs, and pets (Thépault et al., 2017), although chicken is recognized as the main source of human infection in most countries (Domingues et al., 2012; Newell et al., 2017; Cody et al., 2019). Despite consumption of contaminated raw or under-cooked poultry meat being the main source of human infection (Domingues et al., 2012), other sources of infection include contaminated water, unpasteurized/incompletely pasteurized milk, or the direct contact with animals and the environment (Lévesque et al., 2008; de Perio et al., 2013; Levallois et al., 2014; Fernandes et al., 2015; Mossong et al., 2016).

Antimicrobial-based therapy for campylobacteriosis is indicated only in patients with severe and invasive gastroenteritis. Macrolides (mainly erythromycin), fluoroquinolones (mainly ciprofloxacin), and tetracyclines are the most commonly used and effective antibiotics (Aarestrup et al., 2008; Silva et al., 2011). Unfortunately, these antimicrobial agents have analogs widely employed in veterinary settings, and their overuse or misuse have favored the emergence and spread of resistant *Campylobacter* isolates (Van Boeckel et al., 2015; Asuming-Bediako et al., 2019). Furthermore, the continuous increase in resistance to certain antimicrobial classes has been accompanied by a rise in the occurrence of multidrug-resistant (MDR) *C. jejuni* and *C. coli* from farm to fork (Mourkas et al., 2019).

Whole genome sequencing (WGS) is a powerful tool that offers high-resolution sub-typing of *Campylobacter* isolates, which is of value for outbreak investigations (Clark et al., 2016; Lahti et al., 2017). Apart from multilocus sequence typing (MLST) schemes, based on the analysis of the sequence of several house-keeping genes, WGS also allows to predict relevant phenotypes, such as antibiotic resistance, in *Campylobacter* (Zhao et al., 2016). Moreover, recent studies have found high concordance between the presence of antimicrobial resistance determinants in WGS data and phenotypic resistance among *Campylobacter* isolates from Latvia (Meistere et al., 2019), England and Wales (Painset et al., 2020), and Poland (Wysok et al., 2020), supporting the use of WGS data as a good predictor for phenotypical antimicrobial resistance.

Taking into account the increasing relevance of *C. jejuni* and *C. coli* as foodborne pathogens and the availability of a high amount of their genomes in different public repositories, with the corresponding information on the year, country, and source of isolation of the sequenced strains, the objectives of the current study are as follows: (i) to study the host association of the most abundant clonal complexes (CCs) of *C. jejuni* and the most abundant sequence types (ST) within the main CCs on *C. jejuni* and *C. coli*; (ii) to compare the prevalence of antimicrobial resistance (AR) factors within *C. jejuni* and *C. coli* genomes; (iii) to perform an exhaustive resistome genotypic analysis and link AR patterns to country/continent, source of isolation, and *C. jejuni* CCs; and (iv) to assess temporal changes in such AR patterns along the last 20 years.

## Materials and Methods

### Downloading Genomes and Metadata for Each Genome

Metadata associated to each genome was obtained by downloading the table obtained after using “*Campylobacter jejuni*” and “*Campylobacter coli*” as keywords (i) on PATRIC Taxon View website^1^; (ii) through the Export dataset section on the PubMLST-*Campylobacter jejuni/coli* website^2^, where all isolates were selected; and (iii) by using 02.NCBI_Download_metadata.R script^3^ for the NCBI repository. This script basically downloads the information obtained from https://www.ncbi.nlm.nih.gov/biosample/BIOSAMPLE?report=full&format=text, where BIOSAMPLE is substituted by the NCBI Biosample code for each genome analyzed, with these genomes being those of the *C. jejuni* and *C. coli* strains compiled in the prokaryotes.txt file in ftp://ftp.ncbi.nlm.nih.gov/genomes/GENOME_REPORTS/. The three metadata files obtained (for the NCBI, PATRIC, and PubMLST repositories) were manually inspected and adapted to merge them in a unique file. The collection_date, host_name, country, and continent columns were manually inspected to homogenize the collection date (to year of collection), isolation source, and location format (to country and continent).

*C. jejuni* and *C. coli* genomes publicly available at NCBI,^4^ PATRIC,^5^ and PubMLST^6^ repositories were downloaded on February 2021 using the *download_genomes* pipeline made for such task (see footnote 3). Briefly, the lists of available *C. jejuni* and *C. coli* genomes were constructed from metadata files for the PATRIC and PubMLST repositories and from the *prokaryotes.txt* file^7^ for the NCBI repository. Those genomes that were present on more than one repository were discarded from the NCBI or PubMLST dataset using *03.ena2ncbi.rb* and *04.remove_repetition* scripts (see footnote 3), and then, all selected genomes were downloaded using the corresponding script for each database from download_genomes github repository (see footnote 3).

### Genome Analyses

All genomes were analyzed through MLST by using the mlst github pipeline (Seemann), which employs data from the PubMLST.org website (Jolley et al., 2018). Antimicrobial gene detection and detection of single-nucleotide polymorphisms (SNPs) conferring antimicrobial resistance were performed using the ResFinder database version updated at March 12, 2021 (Zankari et al., 2012) and PointFinder database updated at February 1, 2021 (Zankari et al., 2017), respectively. Both analyses, together with MLST, were performed for each genome by using the staramr pipeline (phac-nml/staramr) and *08.staramr_auto.rb* script (see footnote 3) to automatize the analyses for the dozens of thousands genomes analyzed.

### Statistical Analysis

The R-packages *tidyr* and *dplyr* were used to organize the data matrix, while the R-packages *ggplot2* and *pheatmap* were employed for the representation of line plots, boxplots, and heatmap plots using R version 3.6.1 (R Core Team, 2019) and *t*-test to determine statistical differences. Heatmap plots were done using the percentage of genomes carrying each AR determinant (gene or SNP) that belonged to the same isolation source, clonal complex, or isolation year. The Euclidean distance and the complete (= UPGMA) clustering method were employed for the clustering of columns and rows.

Only those isolation sources with more than 50 genomes, excluding the *unknown* group, were selected for obtaining heatmap plots of antibiotic resistance genes (ARGs) or antibiotic families versus isolation source to avoid the introduction of biases due to the low number of genomes. Similarly, European genomes were uniquely selected for temporal variation analyses in the resistome, due to the fact that the highest number of genomes belonged to Europe and North America, but an important proportion of North-American genomes had missing information in relation to the year of isolation. Moreover, only European genomes from isolates obtained in those years when at least 100 genomes were available (i.e., years between 1997 and 2018, with the exception of 1999, 2000, and 2002) were selected for this task.

## Results

### Global Overview

A total of 39,798 *C. jejuni* and 11,920 *C. coli* genomes were analyzed. The *C. jejuni* genomes belonged to 41 CCs, with ST-21 CC being the most abundant (23.8% of the analyzed genomes) followed by ST-353 and ST-45 CCs (8.9 and 7.8% of the analyzed genomes, respectively), while *C. coli* genomes belonged to 21 CCs, with ST-828 CC as the main CC (84.8% of analyzed genomes). Remarkably, 13.7% of the *C. jejuni* and 13.43% of *C. coli* genomes were not assigned to any known CC. The main isolation source was humans (37.1% of *C. jejuni* genomes and 14.8% of *C. coli* genomes), followed by chicken, cows, and sheep for *C. jejuni* (10, 3, and 1.2% of genomes, respectively) and chicken, pigs, cows, and sheep for *C. coli* (9, 5.5, 2.3, and 1.3% of genomes, respectively). Unfortunately, 45.6 and 60.6% of *C. jejuni* and *C. coli* genomes, respectively, had missing information on the isolation source. A total of 18,812 *C. jejuni* (47.3%) and 7,959 *C. coli* (66.8%) genomes were from North-American isolates, while 18,787 *C. jejuni* (47.2%) and 3,328 *C. coli* (27.9%) genomes belonged to European isolates. The genome codes, collection year, continent, country, host and MLST information for all the analyzed *C. jejuni* and *C. coli* genomes were presented to Supplementary Tables 1, 2, respectively.

**FIGURE 1:**
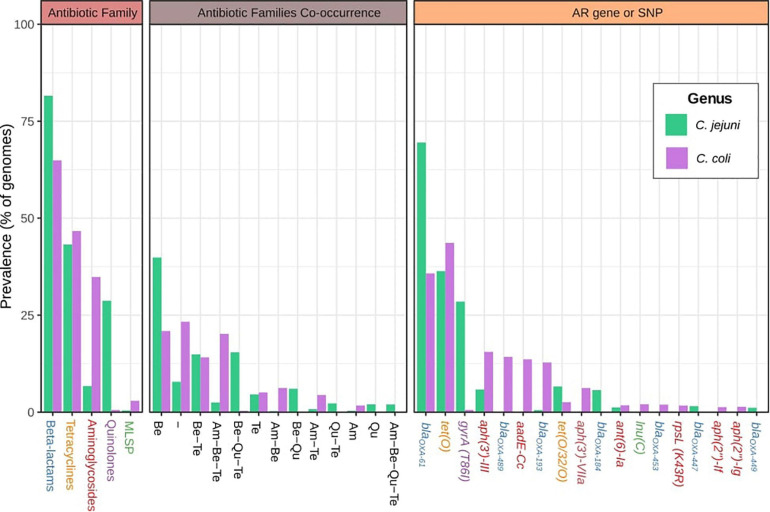
Global antibiotic resistance gene (ARG)-single-nucleotide polymorphism (SNP) distribution on *C. jejuni* and *C. coli* genomes. Antimicrobial resistance families, family co-occurrence, and most abundant ARGs or SNPs among the *C. jejuni* and *C. coli* genomes analyzed. Am, aminoglycosides; Be, beta-lactams; Qu, quinolones; Te, tetracyclines. Gene names are colored according to the corresponding antimicrobial family [blue: beta-lactams, orange: tetracyclines, red: aminoglycosides, purple: quinolones, and green: macrolides-lincosamides-streptogramins-pleuromutilins group (MLSP)]. Hyphen (-) indicates no ARGs or SNPs detected.

The most abundant AR factors were associated with resistance to β-lactams and tetracyclines for both species, although β-lactam resistance was most prevalent on *C. jejuni* (81.6 vs. 65.0%). AR factors against aminoglycosides and antibiotics of the macrolides-lincosamides-streptogramins-pleuromutilins group (MLSP) were most abundant on *C. coli* genomes (34.8 and 2.9%, respectively) than on *C. jejuni* (6.7 and 0.5%, respectively), while resistance to quinolones was much more frequent on *C. jejuni* (28.7%) then on *C. coli* (0.6%) genomes (Figure 1). Moreover, up to 7.8 and 23.3% of *C. jejuni* and *C. coli* genomes, respectively, did not harbor any known AR factor (Figure 1). Remarkably, 39.9 and 21% of *C. jejuni* and *C. coli* genomes, respectively, had only AR genes or SNPs associated with resistance to only β-lactams, while 41.7 and 44.0% of the genomes harbored determinants of resistance to β-lactams combined with determinants of resistance to other antibiotic families. Similar observations were made for resistance to tetracyclines, which were seen as unique resistance factors on 4.6 and 5.1% of *C. jejuni* and *C. coli* genomes, respectively, while 38.6 and 41.6% of genomes harbored resistance to tetracyclines combined with resistance to other antibiotic families (Figure 1). It is worth highlighting the high level of co-occurrence of resistance to aminoglycosides, β-lactams, and tetracyclines on *C. coli* genomes (20.2%) and to β-lactams, quinolones, and tetracyclines on *C. jejuni* genomes (15.4%) (Figure 1).

A total of 120 and 68 different AR genes and SNPs were detected for *C. jejuni* and *C. coli* genomes, respectively. *bla*_*OXA–*61_ and *tet(O)* genes were the most extended AR determinants on both *C. jejuni* and *C. coli* genomes (they were carried by 69.5 and 36.4% of *C. jejuni* and 35.8 and 43.6% of *C. coli* genomes, respectively). The *gyrA* (T86I) SNP was the third most prevalent AR factor on *C. jejuni* genomes (28.5%), while *aph(3′)-III*, *bla*_*OXA–*489_, *aadE-Cc*, and *bla*_*OXA–*193_ were present in more than 10% of the *C. coli* genomes (Figure 1).

Moreover, while 33.48% of *C. jejuni* genomes carried only the *bla_*OXA–*61_* gene, 10.7% simultaneously harbored *bla_*OXA–*61_* and *tet(O)*, and 9.8% had *gyrA* (T86I), *bla_*OXA–*61_*, and *tet(O)* in combination, with these three ARG genotype patterns being the most abundant on *C. jejuni* genomes (Table 1). The presence of only the *bla_*OXA–*61_* gene was observed on 10.4% of the *C. coli* genomes, with it being the main genotype pattern found, followed by only the *bla_*OXA–*489_* gene and *bla_*OXA–*61_* together with *tet(O)*, with 6.9% and 6.6% of prevalence, respectively (Table 1).

**TABLE 1:** Most abundant ARG genotypes on the *C. jejuni* and *C. coli* genomes analyzed, ordered by decreasing relative abundance.

# Pattern	ARG genotype – *C. jejuni*	# ARGs	# Genomes	% Genomes	% Accumulate
1	*bla_*OXA*–61_*	1	13,326	33.48	33.48
2	*bla_*OXA*–61_ - tet(O)*	2	4,259	10.70	44.19
3	*bla_*OXA–61*_ -gyrA* (T86I) - *tet(O)*	3	3,915	9.84	54.02
4	–	0	3,119	7.84	61.86
5	*bla_*OXA–61*_ - gyrA* (T86I)	2	1,944	4.88	66.74
6	*tet(O)*	1	1,793	4.51	71.25
7	*bla_*OXA–61*_ -gyrA* (T86I) - *tet(O/32/O)*	3	1,456	3.66	74.91
8	*bla_*OXA–61*_ - tet(O) - aph(3′)-III*	3	860	2.16	77.07
9	*bla*_*OXA–184*_	1	836	2.10	79.17
10	*gyrA* (T86I)	1	799	2.01	81.18
11	*gyrA* (T86I) - *tet(O)*	2	711	1.79	82.96
12	*bla*_*OXA–447*_	1	529	1.33	84.29
13	*tet(O) - bla_*OXA–184*_*	2	507	1.27	85.57
14	*bla_*OXA–61*_ - tet(O/32/O)*	2	498	1.25	86.82

**# Pattern**	**ARG genotype – *C. coli***	**# ARGs**	**# Genomes**	**% Genomes**	**% Accumulate**

1	-	0	2,780	23.32	23.32
2	*bla*_*OXA–61*_	1	1,236	10.37	33.69
3	*bla*_*OXA–489*_	1	826	6.93	40.62
4	*bla_*OXA–61*_ - tet(O)*	2	784	6.58	47.20
5	*tet(O)*	1	478	4.01	51.21
6	*bla_*OXA–61*_ - tet(O) - aph(3′)-III*	3	417	3.50	54.70
7	*bla*_*OXA–193*_	1	322	2.70	57.41
8	*bla_0XA–193_ - tet(O) - aph(3′)-III*	3	302	2.53	59.94
9	*bla_*OXA–193*_ - tet(O)*	2	284	2.38	62.32
10	*bla_*OXA–61*_ - tet(O) - aadE-Cc*	3	283	2.37	64.70
11	*bla_*OXA–489*_ - tet(O)*	2	269	2.26	66.95
12	*bla_*OXA–61*_ - aph(3′)-VIIa*	2	201	1.69	68.64
13	*tet(O) - aadE-Cc*	2	196	1.64	70.28
14	*bla_*OXA–453*_ - tet(O)*	2	186	1.56	71.84
15	*bla_*OXA–193*_ - tet(O) - aadE-Cc*	3	181	1.52	73.36
16	*bla_*OXA–61*_ - aadE-Cc*	2	173	1.45	74.81
17	*blaOXA-193 - tet(O) - aph(3′)-VIIa*	3	167	1.40	76.21
18	*bla_0XA–489_ - tet(O) - aph(3’)-III*	3	157	1.32	77.53
19	*tet(O) - aph(3′)-III*	2	124	1.04	78.57
20	*tet(O/32/O)*	1	123	1.03	79.60

*Only ARG genotype patterns with relative abundance > 1% are presented.*

### Geographical Distribution of CCs and ARGs-SNPs

**FIGURE 2:**
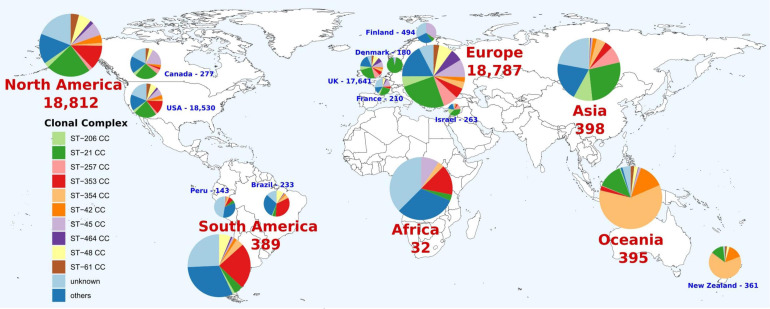
Geographical distribution of *C. jejuni* clonal complexes (CCs). Only countries with more than 100 genomes were represented. The number indicates the genomes available on the corresponding continent or country. Colors within pie charts represent the percentage of genomes belonging to the main CCs, with low abundant CCs grouped as “others” and genomes with unknown CCs as “unknown.”

The first global analysis of *C. jejuni* CC distribution showed differences by continent. The ST-21 CC was the most abundant in Asia, Europe, and North America (26.9, 25.0, and 23.0% of genomes, respectively), while ST-353 CC was predominant in South America and Africa (23.7 and 15.6%, respectively) and ST-354 CC was dominant in Oceania (60.3%) (Figure 1). These patterns are not observed in all the countries from the same continent. For example, ST-353 CC was abundant in the United States and Brazil (12.8 and 33.5% of genomes, respectively), but it was less represented on Canadian and Peruvian genomes (2.2 and 7.0%, respectively), with Canada having a high prevalence of ST-45 CC (18.8%) (Figure 2). The main differences among countries within the same continent were found in Europe, with 93.3% of ST-21 CC genomes in Denmark; 34.4% of ST-45CC genomes in Finland; and 26.7, 25, and 39.5% of ST-21 CC genomes in France, United Kingdom, and Israel, as the main CCs (Figure 2).

**FIGURE 3:**
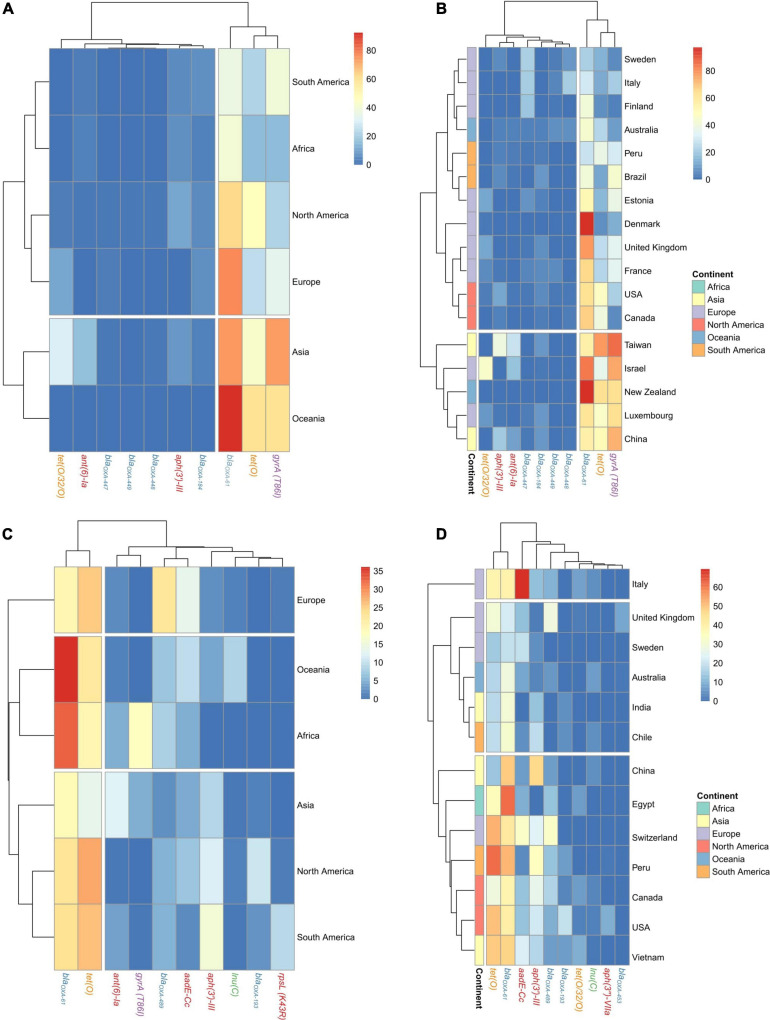
Geographical distribution of AR genes and SNPs on *C. jejuni* and *C. coli* genomes. Heatmap plots showing the distribution of the main AR genes and SNPs on *C. jejuni* genomes among **(A)** continents and **(B)** countries and on *C. coli* genomes among **(C)** continents and **(D)** countries. Countries with less than 20 genomes were discarded for this analysis. Gene names are colored according to the corresponding antimicrobial family (blue: beta-lactams, orange: tetracyclines, red: aminoglycosides, purple: quinolones, and green: MLSP).

The *bla_*OXA–*61_* gene had the highest prevalence among *C. jejuni* genomes in all continents (from 36.5 to 92.4% of total genomes), except for South America, which had the *gyrA* (T86I) SNP as the main AR factor (39.1%) (Figure 3A). Oceania had the highest prevalence of *tet(O)*, followed by North America and Asia (60, 46.9, and 42.7%, respectively), while the *gyrA T86I* SNP had the highest abundance on genomes obtained in Asia and Oceania (73.9 and 59.2%, respectively), which cluster together and separated from those from other continents (Figure 3A and Supplementary Figure 1A). There were some discrepancies at country level, where two main clusters were obtained, the second one containing genomes from Taiwan, Israel, New Zealand, Luxemburg, and China, countries with no geographical or even socio-economical relation, which harbored the highest prevalences of *tet(O)* gene and *gyrA* (T86I) SNP (Figure 3B and Supplementary Figure 1B).

The *tet(O)* and *bla_*OXA–*61_* genes were the most abundant on *C. coli* regardless of the continent, with prevalences ranging from 16.9 to 36.1% and 13.7–28.1% of genomes, respectively. The *aadE-Cc* and *aph(3′)-III* genes showed an important presence on European genomes (22.6 and 13.6%, respectively), while *ant(6’)-Ia* had an important prevalence on North and South American genomes (11.8 and 15.6%, respectively) and the *gyrA (T86I)* SNP on Asian genomes (12.2%) (Figure 3C). At country level, using those countries with at least 20 genomes, the cluster formed by genomes from China, Egypt, Switzerland, Peru, Canada, United States, and Vietnam had a higher prevalence of a*ph(3′)-III*, *bla_*OXA–*61_*, and *tet(O)* genes than the cluster formed by genomes from the United Kingdom, Sweden, Australia, India, and Chile (Figure 3D and Supplementary Figure 1).

### Host Specialization and ARG-SNP Distribution by Source of Isolation

**FIGURE 4:**
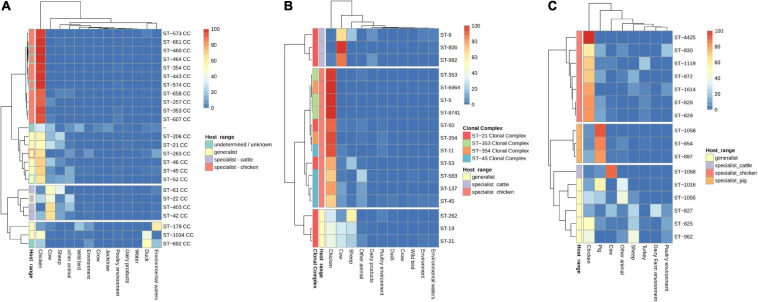
CC and sequence type (ST) host specialization. Heatmap plot showing **(A)** percentage of *C. jejuni* genomes per CC, **(B)** *C. jejuni* genomes per ST within the four main CCs, and **(C)** *C. coli* genomes per ST within ST-828 CC belonging to each isolation source. Genomes belonging to the human isolation source were removed before the calculation of the relative abundances, and CCs/STs with 20 or less genomes were removed to avoid biases due to the low number of genomes. The *Host range* classification of *C. jejuni* CCs is according to previous publications (see Table 1), except for those with highlighted edges, where edge color indicates previous classification while fill color indicates the new assignment, according to the results obtained in the current study.

The analysis of the occurrence of *C. jejuni* CCs in each of the isolation sources, excluding human, showed three clear clusters of CCs. Chicken specialist and generalist CCs formed the biggest group, which was clearly subdivided into two sub-clusters according to host range. Interestingly, chicken was the most frequent source found on the generalist sub-cluster (Figure 4A). Previous studies (French et al., 2005; Griekspoor et al., 2010; Revez et al., 2011; Sheppard et al., 2014; Dearlove et al., 2016; Mourkas et al., 2020) have classified up to eight *C. jejuni* CCs as host generalist and 13 CCs as host specialist, including three CCs specialized in cattle infection, seven in chicken infection, and other three in wild bird infection or environmental persistence (Table 2). According to the clustering obtained, ST-460, ST-464, ST-574, ST-607, and ST-658 CCs, previously not classified according to their host specialist/generalist behavior, can be classified as chicken specialist CCs, while ST-52 CC can be classified as a generalist CC (Figure 4A and Table 2). On the other hand, ST-283 CC, previously described as a specialist chicken CC, was not located within the chicken specialist sub-cluster (Figure 4A), so a change to generalist host range can be proposed according to the data here presented. Another important observed cluster was the cluster of cattle specialist CCs, which also included the ST-22 CC, previously described as generalist (Figure 4A). In this case, the percentage of genomes assigned to the cow source among cattle specialist CCs, which ranged from 44.1 to 66.7% (excluding genomes isolated from human), was not as high as that of genomes assigned to chicken source for chicken specialist CCs, which was in all cases above 90%. In addition, cattle specialist CCs had similar percentages of abundance in the cow source than generalist CCs in the chicken source (Figure 4A and Supplementary Figure 2A). Therefore, it is important to remark the high level of specialization of those CCs considered as chicken specialist, compared to other specialist groups. Moreover, a ST host specialization analysis performed with the most abundant CCs showed that ST-45 CC harbors STs with medium levels of chicken specialization, while ST-353 and ST-354 CCs contain STs highly specialized for chicken (Figure 3B and Supplementary Figure 2B). Finally, the ST-21 CC contains three STs specialized in cattle infection (ST-8, ST-806, and ST-982), two specialized in chicken infection (ST-50 and ST-53), one ST specialized on sheep infection (ST-262), and two generalist STs (ST-19 and ST-21) (Figure 4B and Supplementary Figure 2B). The ST-858 CC, which was the main one found for *C. coli* genomes, harbors up to seven STs (ST-4425, ST-830, ST-1119, ST-872, ST-1614, ST-828, and ST-829) that could be considered as chicken infection specialist, three pig specialized STs (ST-1058, ST-854, and ST-887), one cattle specialist ST (ST-1068), and five host generalist STs (ST-1016, ST-1055, ST-827, ST-825, and ST-962) (Figure 4C and Supplementary Figure 2C). The complete list of STs analyzed, from both *C. jejuni* and *C. coli* genomes, and their host specilization were indicated on Supplementary Table 3.

**TABLE 2:** Clonal complex classification by their host specialization according to previous publications and to the results of the current study.

Species	Clonal complex	# Genomes	# Non-human genomes	Host specialization	References
*C. jejuni*	ST-21 CC	9,477	5,660	Generalist	Sheppard et al., 2014; Dearlove et al., 2016
*C. jejuni*	ST-22 CC	799	497	Generalist *	Revez et al., 2011
*C. jejuni*	ST-4l CC	28	22	–	–
*C. jejuni*	ST-42 CC	1,390	1,024	Specialist_cattle	Sheppard et al., 2014
*C. jejuni*	ST-45 CC	3,103	1,978	Generalist	Sheppard et al., 2014; Dearlove et al., 2016
*C. jejuni*	ST-48 CC	2,667	1,466	Generalist	Sheppard et al., 2014
*C. jejuni*	ST-49 CC	381	280	–	–
*C. jejuni*	ST-52 CC	639	390	Generalist	Present study
*C. jejuni*	ST-61 CC	1,422	1,110	Specialist_cattle	Sheppard et al., 2014; Mourkas et al., 2020
*C. jejuni*	ST-177 CC	26	23	Specialist_wild-birds/envs	Sheppard et al., 2014
*C. jejuni*	ST-179 CC	208	198	Specialist_wild-birds/envs	Sheppard et al., 2014
*C. jejuni*	ST-206 CC	1,570	677	Generalist	Sheppard et al., 2014
*C. jejuni*	ST-257 CC	1,857	797	Specialist_chicken	Sheppard et al., 2014
*C. jejuni*	ST-283 CC	290	127	Specialist_chicken *	Sheppard et al., 2014
*C. jejuni*	ST-353 CC	3,550	2,643	Specialist_chicken	Sheppard et al., 2014
*C. jejuni*	ST-354 CC	1,220	598	Specialist_chicken	Sheppard et al., 2014
*C. jejuni*	ST-362 CC	31	9	–	–
*C. jejuni*	ST-403 CC	533	362	Specialist_cattle	French et al., 2005
*C. jejuni*	ST-433 CC	16	7	–	–
*C. jejuni*	ST-443 CC	905	482	Specialist_chicken	Sheppard et al., 2014
*C. jejuni*	ST-446 CC	55	16	–	–
*C. jejuni*	ST-460 CC	256	180	Specialist_chicken	Present study
*C. jejuni*	ST-464 CC	1,531	679	Specialist_chicken	Present study
*C. jejuni*	ST-508 CC	393	299	–	–
*C. jejuni*	ST-573 CC	216	177	Specialist_chicken	Sheppard et al., 2014
*C. jejuni*	ST-574 CC	326	91	Specialist_chicken	Present study
*C. jejuni*	ST-607 CC	447	320	Specialist_chicken	Present study
*C. jejuni*	ST-658 CC	368	68	Specialist_chicken	Present study
*C. jejuni*	ST-661 CC	117	86	Specialist_chicken	Sheppard et al., 2014
*C. jejuni*	ST-677 CC	145	17	Generalist	Griekspoor et al., 2010
*C. jejuni*	ST-682 CC	9	9	Specialist_wild-birds/envs	Sheppard et al., 2014
*C. jejuni*	ST-692 CC	87	64	–	–
*C. jejuni*	ST–702 CC	18	16	–	–
*C. jejuni*	ST-828 CC	23	14	Generalist	Dearlove et al., 2016
*C. jejuni*	ST-952 CC	23	19	–	–
*C. jejuni*	ST-1034 CC	120	73	Generalist	Griekspoor et al., 2010
*C. jejuni*	ST-1150 CC	1	1	–	–
*C. jejuni*	ST-1275 CC	23	16	–	–
*C. jejuni*	ST-1287 CC	31	20	–	–
*C. jejuni*	ST-1304 CC	6	6	–	–
*C. jejuni*	ST-1332 CC	31	25	–	–
*C. jejuni*	Unknown	5,460	4,472	–	–
*C. coli*	ST-828 CC	10,106	8,590	–	–
*C. coli*	ST-1150 CC	174	161	–	–
*C. coli*	others	39	32	–	–
*C. coli*	Unknown	1,601	1,368	–	–

*Those CCs with less than 20 genomes with a known non-human source of isolation were not employed for the specialist/generalist host association analysis. The asterisk indicates CCs with new classification of host specialism/generalist behavior proposed, according to the data obtained in the current study. ST-22 CC is proposed to be cattle specialist instead of generalist and ST-283 CC to be generalist instead of chicken specialist (see Figure 4).*

**FIGURE 5:**
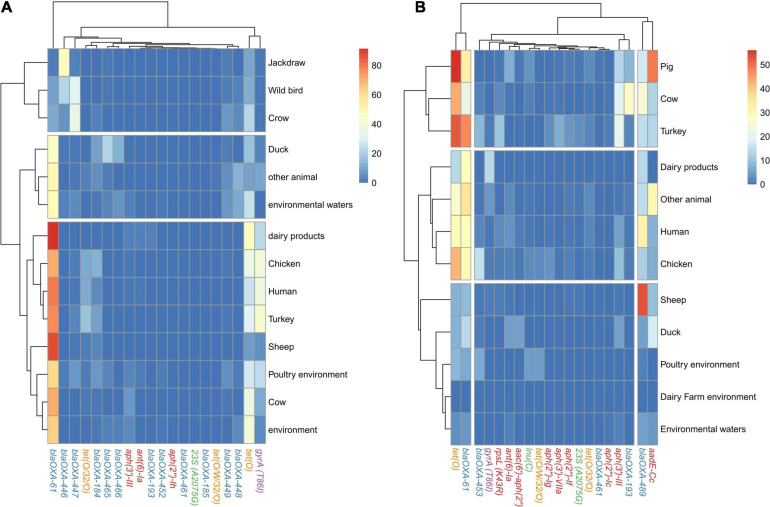
AR genes and SNP distribution among *C. jejuni* and *C. coli* genomes by source of isolation. Heatmap plot showing the percentage of **(A)** *C. jejuni* genomes and **(B)** *C. coli* genomes per isolation source carrying ARGs or SNPs associated with AR, showed in columns. Only the main 20 ARGs or SNPs are presented, because the clustering by isolation source remained the same as when the 120 and 68 AR determinants were used for *C. jejuni* and *C. coli*, respectively (data not shown). Only isolation sources containing more than 50 genomes are presented. Gene names are colored according to the corresponding antimicrobial family (blue: beta-lactams, orange: tetracyclines, red: aminoglycosides, purple: quinolones, and green: MLSP).

The analysis of the occurrence of AR determinants in genomes from each of the isolation sources revealed three main clusters of both *C. jejuni* and *C. coli* genomes. The main cluster of *C. jejuni* genomes related to food production sources, which showed a higher prevalence of *bla_*OXA–*61_* (from 44.7 to 100%) and of the *gyrA* (T86I) SNP (from 1.5 to 41.1%) than genomes from the second cluster obtained [from 0 to 12.84% for *bla_*OXA–*61_*; from 1.3 to 4.6% for the *gyrA* (T86I) SNP] (Figure 5A and Supplementary Figure 3A). This second cluster harbored genomes from isolates coming from host animals not linked to industrialized food production, such as jackdaws, crows, and other wild birds (Figure 5B). The lower prevalence of *bla_*OXA–*61_* and the higher diversity of other genes associated with resistance to β-lactams among the genomes from this second cluster indicate a lower selection pressure to keep this prevalent *bla_*OXA–*61_* gene in non-livestock animal populations. The last cluster of *C. jejuni* genomes was formed by genomes from duck, other animals, and environmental waters and presented an intermediate prevalence of *bla_*OXA–*61_* compared to the other two clusters obtained (Figure 5A and Supplementary Figure 3A). Similar clusters were found for *C. coli* genomes, with pig, cow, and turkey forming the cluster with the highest prevalence of *tet(O)* and *bla_*OXA–*61_*, with *tet(O)* being more prevalent than on *C. jejuni* genomes while *bla_*OXA–*61_* was less prevalent (Figure 5B and Supplementary Figure 3B). A second cluster of *C. coli* genomes was formed by genomes from dairy products, humans, chicken, and other animals and was characterized by an intermediate prevalence of *tet(O)* and *bla_*OXA–*61_* genes, compared to the other clusters (Figure 5B and Supplementary Figure 3B). Finally, the third cluster, with the lowest prevalence of AR determinants, was formed by genomes from environmental samples, sheep, and duck. It is important to highlight the high prevalence of *aadE-Cc* on genomes from pigs and *bla_*OXA–*489_* on genomes from sheep (Figure 5B).

### ARG-SNP Distribution Among *C. jejuni* CCs

**FIGURE 6:**
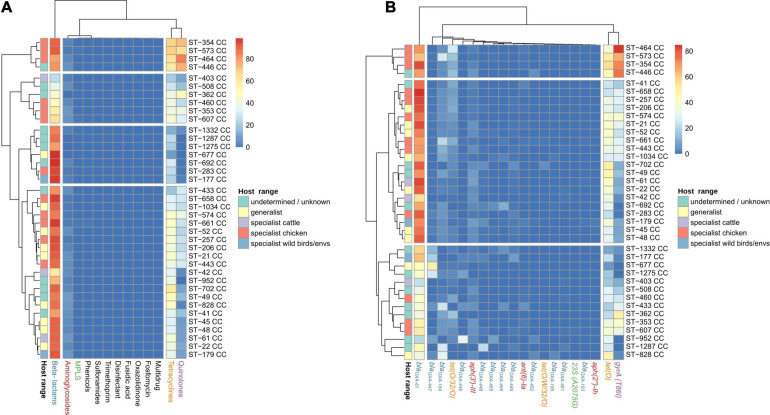
AR genes and SNP distribution among *C. jejuni* CCs. Heatmap plot showing the percentage of genomes per CC carrying **(A)** ARGs or SNPs associated with resistance to antibiotic families and **(B)** particular ARGs or SNPs, showed in columns. Only the main 20 ARGs or SNPs are presented, because the clustering by isolation source remained the same as when the 120 AR determinants were used (data not shown). The *Host_range* classification of CCs is according to previous publications (see Table 1), except for those with highlighted edges, where edge color indicates previous classification while fill color indicates the new assignment, according to the results showed in Figure 4. Gene names are colored according to the corresponding antimicrobial family (blue: beta-lactams, orange: tetracyclines, red: aminoglycosides, purple: quinolones, and green: MLSP).

Up to four main clusters were obtained in the analyses performed at antibiotic family level. Cluster 1 harbored four CCs with the highest prevalence of determinants of resistance to tetracyclines and quinolones, while cluster 2, formed by 20 CCs, had the second main prevalence for tetracycline and quinolone resistance determinants (Figure 6A and Supplementary Figure 4A). Cluster 3, formed by seven CCs, had the lowest prevalence of quinolone and tetracycline resistance determinants (Figure 6A and Supplementary Figure 4A), and cluster 4, formed by six CCs, had the lowest abundance of β-lactam resistance determinants (Figure 6A and Supplementary Figure 4A). At gene level, three clusters were found: cluster 1, harboring four CCs with a high prevalence of *gyrA* (T86I) SNP, *bla_*OXA–*61_*, and *tet(O)*; cluster 2, composed of 20 CCs with a high prevalence of *bla_*OXA–*61_*; and cluster 3, with 14 CCs showing the lowest prevalence of *gyrA* (T86I) SNP, *bla_*OXA–*61_*, and *tet(O)* (Figure 6B and Supplementary Figure 4B). No clear association between genotype patterns at antibiotic family level and host specialist/generalist status was observed. As an example, cluster 1, characterized by a high prevalence of ARGs linked to resistance to tetracyclines and quinolones, was mainly formed by chicken specialist CCs, but other chicken specialist CCs were located across the other three remaining clusters (Figure 6A). A similar absence of a clear association with the host range of each CC was observed at gene level.

### Temporal Changes of AR Determinants on Europe

**FIGURE 7:**
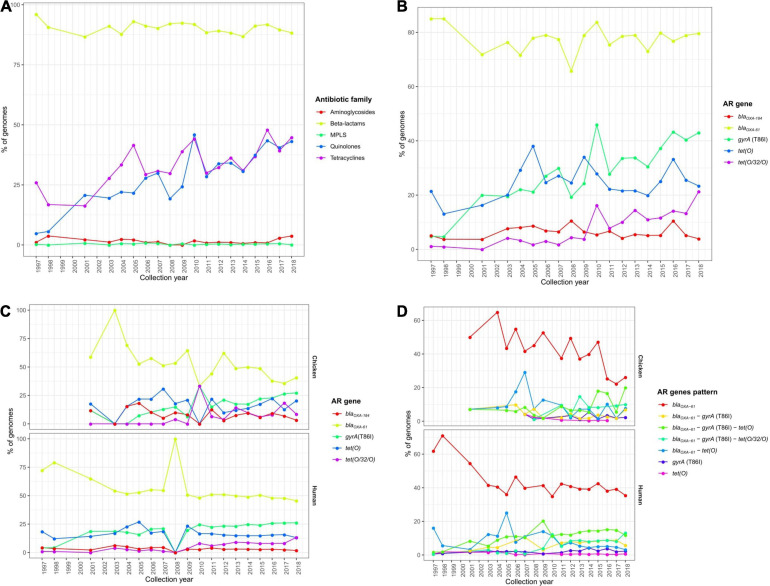
Temporal changes in the resistome of European *C. jejuni* genomes. Prevalence of **(A)** ARGs or SNPs associated with resistance to different antibiotic families and **(B)** the main ARGs or SNPs found on all European *C. jejuni* genomes and **(C)** main ARGs or SNPs and **(D)** AR patterns on *C. jejuni* genomes from chicken and human sources along the last 20 years.

Focusing on temporal changes in the repertoire of AR determinants among the *C. jejuni* European genomes, there was a clear stability in the occurrence of β-lactam ARGs along time (with mean prevalence values per each 5-year period ranging from of 88.8 to 93.3%), while an important increase was observed in the prevalence of determinants of resistance to tetracyclines, from a mean value of 29.8% in the 2000–2004 5-year period to 44.0% in the 2015–2018 period, and quinolones, from 21.0% in the 2000–2004 5-year period to 42.4% in the 2015–2018 period (Figure 7A and Supplementary Figure 5A).

These observations were linked to an increase in the occurrence of both the *tet(O)* and *tet(O/W/O)* genes and the *gyrA* (T86I) SNP (Figure 7B and Supplementary Figure 5B), which were the main determinants of resistance to tetracyclines and quinolones, respectively. Furthermore, the increases along time in the *tet(O/W/O)* gene and the *gyrA* (T86I) SNP were observed in both human and chicken genomes (Figure 7C and Supplementary Figures 5C,D). Finally, the enhanced occurrence over time of resistance to tetracyclines and quinolones was mainly correlated with a significant increase in *bla_*OXA–*61_*–*tet(O)–gyrA* (T86I) and *bla_*OXA–*61_*–*tet(O/W/O)–gyrA* (T86I) multi-drug resistance genotype patterns on *C. jejuni* genomes obtained from human and chicken, which showed mean prevalence values of 10.5 and 6.1% in the 2000–2004 5-year period and 25.6 and 29.9% in the 2015–2018 period, respectively, representing a significant increase in co-occurrence of such genes (Figure 7D and Supplementary Figures 5E,F).

**FIGURE 8:**
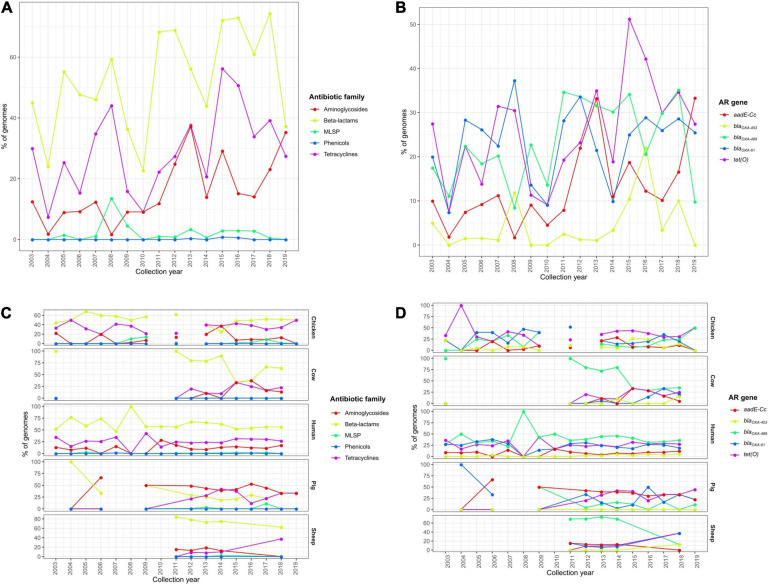
Temporal changes in the resistome of European *C. coli* genomes. Prevalence of **(A)** ARGs or SNPs associated with resistance to different antibiotic families and **(B)** the main ARGs or SNPs found on all European *C. coli* genomes and **(C)** the main ARGs or SNPs and **(D)** AR patterns on *C. jejuni* genomes from chicken, cow, human, pig, and sheep sources along the last 20 years.

Similar temporal analyses were performed on *C. coli* genomes, but no clear trends of AR determinant increase along time were detected. The observed slight increases in the prevalence of determinants of aminoglycoside and tetracycline resistance (Figure 8A), highly correlated with an increase of *addE-Cc* and *tet(O)* prevalence (Figure 8B), were associated with a differential prevalence on genomes from cows and pigs, with more *C. coli* genomes being sequenced on the last years (Figures 8C,D).

## Discussion

At a first global view, the distribution of *C. jejuni* CCs was different among continents, with ST-21 CC as the main one in North America, Europe, and Asia; ST-353 CC in South America and Africa, and ST-354 CC in Oceania. Moreover, this distribution by continent was not maintained among all the countries within a given continent, i.e., Denmark harbored a strong dominance of ST-21 CC genomes while Finland had ST-45 CC as the most abundant CC. Surprisingly, a lack of main association within developing–developed countries and AR composition on *C. jejuni* and *C. coli* genomes was clearly found, maybe due to the unequal distribution of both CC-STs and host origin of isolation among countries and even the amount of sequenced genomes for each country.

Results obtained in the current study reveal the association of certain *C. jejuni* CCs and both *C. jejuni* and *C. coli* STs to particular isolation sources. In this regard, it is worth mentioning the high abundance of CCs linked to chicken as a host. Most of these *C. jejuni* CCs had been already classified as a chicken specialist (Sheppard et al., 2014), and our study confirmed these previous associations, while ST-283 CC, previously described as a chicken specialist (Sheppard et al., 2014), can be considered as a generalist according to our findings. Moreover, among previously not assigned CCs, ST-460, ST-464, ST-574, ST-607, and ST-658 CCs can be considered as a chicken specialist, ST-52 CC as a generalist, and ST-22 CC as a cattle specialist, according to the results here reported. The high abundance of genomes from generalist ST-21 and chicken specialist ST-353 CCs agrees with previous reports that have described these CCs as highly prevalent among clinical, food, and animal samples from Israel (Rokney et al., 2018); clinical cases, dairy cattle, and broiler products in Lithuania (Ramonaite et al., 2017); chicken in Senegal (Kinana et al., 2006); or human and broiler carcasses in Belgium (Elhadidy et al., 2018). The high abundance of ST-21, ST-48, ST-45, and ST-257 CCs on isolates obtained from human samples has been previously highlighted in the United Kingdom (Haldenby et al., 2020). Interestingly, the STs within ST-45 CC, previously considered as a generalist CC, could be considered as chicken specialist STs. Conclusions on host specialization have to be taken carefully. For example, the generalist ST-21 CC harbored STs considered as chicken specialist (ST-50 and ST-53), cow specialist (ST-8, ST-806, and ST-982), and generalist (ST-19, ST-21, and ST-262). This host specialization differences within ST-21 CC could be due to the paraphyletic phylogeny of this CC, which was found as the ancestor CC of the cattle specialist ST-61 CC (Sheppard et al., 2014; Mourkas et al., 2020). The prevalence of ST-353, ST-354, ST-50, and ST-45 on chicken hosts and ST-21 as a host generalist were previously reported by using all the profiles on PubMLST (Pascoe et al., 2020), which are twice the number of genomes available on that web repository and employed in the current study. That supports the proposed host specialization classification, despite the possible biases introduced due to the isolates selected or employed for whole genome sequencing within all the available collections of bacterial isolates.

Determinants of resistance to β-lactams and tetracyclines were the most extended among the 39,798 *C. jejuni* and 11,920 *C. coli* genomes analyzed, with *bla_*OXA–*61_* as the main β-lactam resistance factor on *C. jejuni*; *bla_*OXA–*61_*, *bla_*OXA–*489_*, *and bla_*OXA–*193_* on *C. coli*; and *tet(O)* for tetracycline resistance on both species. Determinants of resistance to quinolones [due mainly to the *gyrA* (T86I) SNP] and to aminoglycosides [by *aph(3′)-III*, *aadE-Cc*, and *aph(3′)-VIIa* genes, among others) were also highly prevalent on *C. jejuni* and *C. coli* genomes, respectively. Phenotypic resistance to β-lactam antibiotics in *C. jejuni* is frequently linked to the production of β-lactamase enzymes, encoded by *bla*_*OXA*_ genes (Jonker and Picard, 2010), while tetracycline and quinolone resistance has been often associated with the *tet(O)* gene (Meistere et al., 2019) and the *gyrA* (T86I) SNP, respectively (Kinana et al., 2006; Meistere et al., 2019; Abraham et al., 2020; Joensen et al., 2020). Indeed, a high prevalence of *bla_*OXA–*61_* and *tet(O)* has been found on *C. jejuni* isolated from human, chicken, and cattle in Spain in a study where SNPs were not screened (Mourkas et al., 2019) and on *Campylobacter* spp. in a global study analyzing 237 genomes from the RefSeq NCBI database (Rivera-Mendoza et al., 2020). Moreover, AR determinants of aminoglycoside resistance on *C. coli* and quinolone resistance on *C. jejuni* were mainly found on genomes associated with multidrug resistance, together with AR determinants for β-lactams and tetracyclines.

The increase in resistance to quinolones on *Campylobacter* isolates during the last years has been reported worldwide, including the United States (Nachamkin et al., 2002), China (Wang et al., 2016), France (Gallay et al., 2007), Vietnam (Nguyen et al., 2016), and Peru (Pollett et al., 2012), among other countries, and the usage of fluoroquinolones in poultry farms has been associated with an increase in resistance to quinolones in chicken and human *Campylobacter* isolates (Wieczorek and Osek, 2013). An increased occurrence of determinants of resistance to β-lactams, tetracyclines, and quinolones was found among those *C. jejuni* and *C. coli* genomes from human and food animal origin. The clear differences observed in the resistome between *Campylobacter* genomes from animals employed for food production and those from non-livestock animals can be likely related to the selective pressure exerted by antibiotic use in veterinary settings. Isolates from livestock species were characterized by a higher incidence of *bla_*OXA–*61_*, associated with resistance to β-lactam antibiotics, such as amoxicillin or ampicillin, antibiotics that are frequently employed for veterinary uses. Quinolones and tetracyclines are still available for treatment of livestock all over the world (WHO, 2017), so this is likely the main cause of the resistance levels found for such antibiotics on isolates from food-related animals. Antibiotic resistance represents a great concern mainly in livestock animals due to the misuse of antibiotics. As an example, erythromycin (macrolide), ampicillin (β-lactam), tetracycline (tetracycline), nalidixic acid, and ciprofloxacin (both belonging to the quinolones family) are the drugs toward which the most resistant isolates were found in a recent review on *Campylobacter* spp. in Sub-Saharan African countries (Hlashwayo et al., 2020), which is in agreement with results found globally or also with the temporal changes among European isolates described in the current study.

Although the current study does not involve a source attribution analysis, the similar ARG genotype patterns and levels of prevalence of *bla_*OXA–*61_*, *tet(O)*, and *gyrA* (T86I) found among human, chicken, and turkey *C. jejuni* genomes, and of *bla_*OXA–*61_* and *tet(O)* among human and chicken *C. coli* genomes, suggest a high level of relatedness among isolates from these origins. Poultry has been reported as the main source of human infection in studies carried out in different locations including Denmark (Joensen et al., 2020), Sub-Saharan Africa (Gahamanyi et al., 2020), or Lithuania (Ramonaite et al., 2017). On the other hand, other authors have identified as important sources of human infection both chicken and ruminants in France (Berthenet et al., 2019), the United States (Kelley et al., 2020), Israel (Rokney et al., 2018), and Sub-Saharan Africa (Hlashwayo et al., 2020) or chicken and wild birds in Baltic countries (Mäesaar et al., 2020). Moreover, the detection of no major differences in the resistance profiles among isolates from different points of the chicken meat processing chain (Dramé et al., 2020) suggests that AR spread is originated on upstream steps, such as the animal feeding, where the animals are exposed to antibiotics. A clear link between human and chicken *Campylobacter* isolates has been previously demonstrated by combining pulsed-field gel electrophoresis (PFGE) to subtype strains and antibiotic resistance phenotypic testing (Zhao et al., 2015).

The spread of AR genes among *Campylobacter* isolates from humans, animals, and the environment has been reported previously (Mourkas et al., 2019), and it should explain the presence of high levels of ARGs conferring resistance to tetracyclines in human isolates despite these antibiotics are not clinically employed in human medicine. Moreover, tetracycline is broadly employed in the poultry industry worldwide (Van Boeckel et al., 2015), which could be an important reservoir for resistant strains. Furthermore, higher levels of resistant *C. jejuni* and *C. coli* isolates were observed among isolates from pigs than from wild boars in Italy (Marotta et al., 2020), where an important amount of multidrug-resistant strains were isolated from pigs, being the over-use of antibiotics the main cause of the spread of multidrug resistance among pig isolates.

The *bla_*OXA–*61_* gene is highly prevalent among *Campylobacter* spp. isolated from poultry (Griggs et al., 2009). The high diversity and abundance of determinants of resistance to β-lactams different to *bla_*OXA–*61_* among isolates from non-food-related animals and environments could be due to the ability of *Campylobacter* spp. for intrinsic production of β-lactamases in the absence of selective pressure (Cody et al., 2012). The *bla_*OXA–*61_* gene, chromosomally encoded (Rivera-Mendoza et al., 2020), can generate many variants with few SNPs, differing from other *bla*_*oxa*_-encoded proteins in only one (e.g., *bla_*OXA–*193_*) or two amino acids (e.g., *bla_*OXA–*489_*). The *bla_*OXA–*61_* gene and aminoglycoside resistance genes are confined to *C. jejuni* and *C. coli* genomes within *Campylobacter* species, while *gyrA* T86I SNP was mostly presented on *C. jejuni* (Rivera-Mendoza et al., 2020). Unfortunately, ACT to ATT mutation on *gyrA* position 86, the most common on *C. coli* strains with resistance to quinolones (Zirnstein et al., 2000), was not included on PointFinder database, which includes ACA to ATA mutation, the most prevalent on *C. jejuni*. So that, the prevalence of quinolone resistance among *C. coli* genomes found on the present study can be due to this technical issue.

The clonal complexes ST-354, ST-573, ST-464, and ST-446, the first three considered chicken specialist, were the CCs with the highest prevalence of ARGs linked to β-lactam, tetracycline, and quinolone resistance, due to the presence of the *bla_*OXA–*61_*, *tet(O)*, and *gyrA* (T86I) determinants for all of them. Some of these CCs (ST-354, ST-446, and ST-464) have been previously associated with ciprofloxacin (quinolone) resistance in human isolates (Cody et al., 2012). ARGs were more randomly distributed among CCs than among isolation sources. Hence, ARG genotype patterns were more closely related to the source of isolation (and probably the use of antibiotics) than to the lineage of the strains, as also remarked previously (Cody et al., 2012).

An increase in the occurrence of genotypes of resistance to tetracyclines and quinolones, mainly due to the spread of *bla_*OXA–*61_*–*gyrA* (T86I)–*tet(O)* and *bla_*OXA–*61_–gyrA (T86I)–tet(O/W/O)* multi-drug resistance genotypes, was observed along the last 20 years in European *C. jejuni* genomes. The main reason for this temporal trend could be the continuous misuse of tetracyclines and quinolones, still broadly employed on livestock all over the world (WHO, 2017). Interestingly, fluoroquinolones are not utilized in Australian livestock, where *Campylobacter* isolates from chicken meat show very scarce levels of resistance to fluoroquinolones, always due to *gyrA* (T86I) SNPs (Abraham et al., 2020). On the other hand, an increase over time in the prevalence of the *gyrA* (T86I) SNP among isolates of *C. jejuni* associated with human gastrointestinal disease has been reported in the United Kingdom from 1993–1996 to 2008–2009 and even to 2016–2017 (Haldenby et al., 2020). This spread of resistance to quinolones is an important concern in some countries, such as Latvia, where resistance to quinolones was found, due to *gyrA* (T86I), in 90% of the *C. jejuni* isolates obtained from clinical and food-related sources from 2008 to 2016 (Meistere et al., 2019). Remarkably, it has been demonstrated that high levels of fluoroquinolone resistance can persist in poultry even after discontinued use of these antibiotics (Price et al., 2005) and that resistance can rapidly emerge in poultry flocks (Humphrey et al., 2005). Some fitness benefits linked to the *gyrA* (T86I) SNP could be the reason for such high resistance prevalence in settings where the use of fluoroquinolones has been reduced, as has been proposed by Haldenby et al. (2020). Another example of temporal increases in fluoroquinolone resistance among *Campylobacter* species was observed on fecal samples from cattle in France between 2002 and 2006, with the fluoroquinolone resistance rate rising from 29.7% to 70.4% (Châtre et al., 2010).

In conclusion, the current work represents the biggest study to date, as far as we know, of the resistome markers harbored in *C. jejuni/coli* genomes, with 39,978 *C. jejuni* and 11,920 *C. coli* genomes *in silico* screened to search both for antimicrobial resistance genes and SNPs conferring antimicrobial resistance. The results obtained suggest the association between antimicrobial use in veterinary settings, in particular in poultry production, and the spread of resistance to Be, Qu, and Te and evidence the rapid expansion in Europe in the last two decades of determinants of resistance to these antibiotic families, especially among poultry and human isolates. The workflow employed is available to be used in future global resistome analyses for other species of interest, or even to be adapted to other kinds of microbiome analyses.

## Data Availability Statement

The original contributions presented in the study are included in the article/Supplementary Material, further inquiries can be directed to the corresponding author.

## Author Contributions

AÁ-O and JC-D created the study design. JC-D and PG wrote and employed the Ruby and R scripts and downloaded genomes, made data analysis, and plotted the figures. JC-D and PG wrote the first draft of the manuscript and AÁ-O revised it. All authors revised the manuscript and approved the final version.

## Conflict of Interest

The authors declare that the research was conducted in the absence of any commercial or financial relationships that could be construed as a potential conflict of interest.

## Footnotes

1 https://patricbrc.org/view/Taxonomy/2#view_tab=genomes

2 https://pubmlst.org/bigsdb?db=pubmlst_Campylobacter_isolates&page=plugin&name=Export

3 https://github.com/JoseCoboDiaz/download_genomes

4 ftp://ftp.ncbi.nlm.nih.gov/genomes/

5 ftp://ftp.patricbrc.org/

6 https://pubmlst.org

7 ftp://ftp.ncbi.nlm.nih.gov/genomes/GENOME_REPORTS/prokaryotes.txt

## References

[B1] Aarestrup F. M. McDermott P. F. Wegener H. C. (2008). *Transmission of Antibiotic Resistance from Food Animals to Humans*, 3rd Edn. Washington, DC: American Society for Microbiology. 10.1128/9781555815554

[B2] Abraham S. Sahibzada S. Hewson K. Laird T. Abraham R. Pavic A. (2020). Emergence of fluoroquinolone-resistant *Campylobacter jejuni* and *Campylobacter coli* among Australian chickens in the absence of fluoroquinolone use. *Appl. Environ. Microbiol.* 86:2765. 10.1128/AEM.02765-19 32033955 PMC7117913

[B3] Asuming-Bediako N. Kunadu A. P.-H. Abraham S. Habib I. (2019). *Campylobacter* at the human-food interface: the african perspective. *Pathogens* 8:87. 10.3390/pathogens8020087 31242594 PMC6631673

[B4] Berthenet E. Thépault A. Chemaly M. Rivoal K. Ducournau A. Buissonnière A. (2019). Source attribution of *Campylobacter jejuni* shows variable importance of chicken and ruminants reservoirs in non-invasive and invasive French clinical isolates. *Sci. Rep.* 9:8098. 10.1038/s41598-019-44454-2 31147581 PMC6542803

[B5] Bronnec V. Turoòová H. Bouju A. Cruveiller S. Rodrigues R. Demnerova K. (2016). Adhesion, biofilm formation, and genomic features of *Campylobacter jejuni* bf, an atypical strain able to grow under aerobic conditions. *Front. Microbiol.* 7:1002. 10.3389/fmicb.2016.01002 27446042 PMC4927563

[B6] Châtre P. Haenni M. Meunier D. Botrel M. A. Calavas D. Madec J. Y. (2010). Prevalence and antimicrobial resistance of *Campylobacter jejuni* and *Campylobacter coli* isolated from cattle between 2002 and 2006 in France. *J. Food. Prot.* 73 825–831. 10.4315/0362-028x-73.5.825 20501032

[B7] Clark C. G. Berry C. Walker M. Petkau A. Barker D. O. R. Guan C. (2016). Genomic insights from whole genome sequencing of four clonal outbreak *Campylobacter jejuni* assessed within the global *C. jejuni* population. *BMC Genomics* 17:990. 10.1186/s12864-016-3340-8 27912729 PMC5135748

[B8] Cody A. J. McCarthy N. M. Wimalarathna H. L. Colles F. M. Clark L. Bowler I. C. J. W. (2012). A longitudinal 6-year study of the molecular epidemiology of clinical *Campylobacter* isolates in Oxfordshire, United Kingdom. *J. Clin. Microbiol.* 50 3193–3201. 10.1128/JCM.01086-12 22814466 PMC3457434

[B9] Cody A. J. Maiden M. C. J. Strachan N. J. C. McCarthy N. D. (2019). A systematic review of source attribution of human campylobacteriosis using multilocus sequence typing. *Eurosurveillance* 24:1800696. 10.2807/1560-7917.ES.2019.24.43.1800696 31662159 PMC6820127

[B10] Dearlove B. Cody A. Pascoe B. Méric G. Wilson D. J. Sheppard S. K. (2016). Rapid host switching in generalist *Campylobacter* strains erodes the signal for tracing human infections. *ISME J.* 10 721–729. 26305157 10.1038/ismej.2015.149 PMC4677457

[B11] de Perio M. A. Niemeier R. T. Levine S. J. Gruszynski K. Gibbins J. D. (2013). *Campylobacter* infection in poultry-processing workers, Virginia, USA, 2008 -2011. *Emerg. Infect. Dis.* 19 286–288. 10.3201/eid1902.121147 23347390 PMC3559056

[B12] Domingues A. R. Pires S. M. Halasa T. Hald T. (2012). Source attribution of human campylobacteriosis using a meta-analysis of case-control studies of sporadic infections. *Epidemiol. Infect.* 140 970–981. 10.1017/S0950268811002676 22214729

[B13] Dramé O. Leclair D. Parmley E. J. Deckert A. Ouattara B. Daignault D. (2020). Antimicrobial resistance of *Campylobacter* in broiler chicken along the food chain in Canada. *Foodborne Path. Dis.* 17 512–520. 10.1089/fpd.2019.2752 32130036 PMC7415884

[B14] Elhadidy M. Arguello H. Álvarez-Ordóñez A. Miller W. G. Duarte A. Martiny D. (2018). Orthogonal typing methods identify genetic diversity among Belgian *Campylobacter jejuni* strains isolated over a decade from poultry and cases of sporadic human illness. *Int. J. Food Microbiol.* 275 66–75. 10.1016/j.ijfoodmicro.2018.04.004 29649751

[B15] European Food Safety Authority (2018). The European Union summary report on trends and sources of zoonoses, zoonotic agents and food-borne outbreaks in 2017. *EFSA J.* 16:5500. 10.2903/j.efsa.2018.5500 32625785 PMC7009540

[B16] Fernandes A. M. Balasegaram S. Willis C. Wimalarathna H. M. L. Maiden M. C. McCarthy N. D. (2015). Partial failure of milk pasteurization as a risk for the transmission of *Campylobacter* from cattle to humans. *Clin. Infect. Dis.* 61 903–909. 10.1093/cid/civ431 26063722 PMC4551004

[B17] Fischer Walker C. L. Rudan I. Liu L. Nair H. Theodoratou E. Bhutta Z. A. (2013). Global burden of childhood pneumonia and diarrhoea. *Lancet* 381 1405–1416. 10.1016/S0140-6736(13)60222-6 23582727 PMC7159282

[B18] French N. Barrigas M. Brown P. Ribiero P. Williams N. Leatherbarrow H. (2005). Spatial epidemiology and natural population structure of *Campylobacter jejuni* colonizing a farmland ecosystem. *Environ. Microbiol.* 7 1116–1126. 10.1111/j.1462-2920.2005.00782.x 16011749

[B19] Gahamanyi N. Mboera L. Matee M. I Mutangana D. Komba E. (2020). Prevalence, risk factors, and antimicrobial resistance profiles of thermophilic *Campylobacter* species in humans and animals in Sub-Saharan Africa: a systematic review. *Int. J. Microbiol.* 2020:2092478. 10.1155/2020/2092478 32025233 PMC6983289

[B20] Gallay A. Prouzet-Mauleon V. Kempf I. Lehours P. Labadi L. Camou C. (2007). *Campylobacter* antimicrobial drug resistance among humans, broiler chickens, and pigs, France. *Emerg. Infect. Dis.* 13 259–266. 10.3201/eid1302.060587 17479889 PMC2725848

[B21] Griekspoor P. Engvall E. O. Olsen B. Waldenström J. (2010). Multilocus sequence typing of *Campylobacter jejuni* from broilers. *Vet. Microbiol.* 140 180–185. 10.1016/j.vetmic.2009.07.022 19733453

[B22] Griggs D. J. Peake L. Johnson M. M. Ghori S. Mott A. Piddock L. J. V. (2009). β-lactamase-mediated β-lactam resistance in *Campylobacter* species: prevalence of Cj0299 (*blaOXA-61*) and evidence for a novel β-lactamase in *C. jejuni*. *Antimicrob. Agents Chemother.* 53 3357–3364. 10.1128/AAC.01655-08 19506058 PMC2715628

[B23] Haldenby S. Bronowski C. Nelson C. Kenny J. Martinez-Rodriguez C. Chaudhuri R. (2020). Increasing prevalence of a fluoroquinolone resistance mutation amongst *Campylobacter jejuni* isolates from four human infectious intestinal disease studies in the United Kingdom. *PLoS One* 15:e0227535. 10.1371/journal.pone.0227535 31999701 PMC6992184

[B24] Hlashwayo D. F. Sigaúque B. Bila C. G. (2020). Epidemiology and antimicrobial resistance of *Campylobacter* spp. in animals in Sub-Saharan Africa: a systematic review. *Heliyon* 6:e03537. 10.1016/j.heliyon.2020.e03537 32181402 PMC7063338

[B25] Humphrey T. J. Jørgensen F. Frost J. A. Wadda H. Domingue G. Elviss N. C. (2005). Prevalence and subtypes of ciprofloxacin-resistant *Campylobacter* spp. in commercial poultry flocks before, during, and after treatment with fluoroquinolones. *Antimicrob. Agents Chemother.* 49 690–698. 10.1128/AAC.49.2.690-698.2005 15673753 PMC547194

[B26] Joensen K. G. Kiil K. Gantzhorn M. R. Nauerby B. Engberg J. Holt H. M. (2020). Whole-genome sequencing to detect numerous *Campylobacter jejuni* outbreaks and match patient isolates to sources, Denmark, 2015-2017. *Emerg. Infect. Dis.* 26 523–532. 10.3201/eid2603.190947 32091364 PMC7045838

[B27] Jolley K. A. Bray J. E. Maiden M. C. J. (2018). Open-access bacterial population genomics: BIGSdb software, the PubMLST.org website and their applications. *Wellcome Open Res.* 3:124. 10.12688/wellcomeopenres.14826.1 30345391 PMC6192448

[B28] Jonker A. Picard J. A. (2010). Antimicrobial susceptibility in thermophilic *Campylobacter* species isolated from pigs and chickens in South Africa. *J. S. Afr. Vet. Assoc.* 81 228–236. 10.4102/jsava.v81i4.153 21526738

[B29] Kaakoush N. O. Castaño-Rodríguez N. Mitchell H. M. Man S. M. (2015). Global epidemiology of *Campylobacter* infection. *Clin. Microbiol. Rev.* 28 687–720. 10.1128/CMR.00006-15 26062576 PMC4462680

[B30] Kelley B. R. Ellis J. C. Large A. Schneider L. G. Jacobson D. Johnson J. G. (2020). Whole-Genome Sequencing and bioinformatic analysis of environmental, agricultural, and human *Campylobacter jejuni* isolates from East Tennessee. *Front. Microbiol.* 11:571064. 10.3389/fmicb.2020.571064 33224113 PMC7674308

[B31] Kinana A. D. Cardinale E. Tall F. Bahsoun I. Sire J.-M. Garin B. (2006). Genetic diversity and quinolone resistance in *Campylobacter jejuni* isolates from poultry in Senegal. *Appl. Environ. Microbiol.* 72 3309–3313. 10.1128/AEM.72.5.3309-3313.2006 16672471 PMC1472360

[B32] Lahti E. Rehn M. Ockborn G. Hansson I. Ågren J. Engvall E. O. (2017). Outbreak of campylobacteriosis following a dairy farm visit: confirmation by genotyping. *Foodborne Pathog. Dis.* 14 326–332. 10.1089/fpd.2016.2257 28350214

[B33] Levallois P. Chevalier P. Gingras S. Dery P. Payment P. Michel P. (2014). Risk of infectious gastroenteritis in young children living in Quebec rural areas with intensive animal farming: results of a case-control study (2004 -2007). *Zoonoses Public Health* 61 28–38. 10.1111/zph.12039 23406420 PMC7165781

[B34] Lévesque S. Frost E. Arbeit R. D. Michaud S. (2008). Multilocus sequence typing of *Campylobacter jejuni* isolates from humans, chickens, raw milk, and environmental water in Quebec, Canada. *J. Clin. Microbiol.* 46 3404–3411. 10.1128/JCM.00042-08 18701662 PMC2566118

[B35] Liu L. Oza S. Hogan D. Perin J. Rudan I. Lawn J. E. (2015). Global, regional, and national causes of child mortality in 2000-13, with projections to inform post-2015 priorities: an updated systematic analysis. *Lancet* 385 430–440. 10.1016/S0140-6736(14)61698-6 25280870

[B36] Mäesaar M. Tedersoo T. Meremäe K. Roasto M. (2020). The source attribution analysis revealed the prevalent role of poultry over cattle and wild birds in human campylobacteriosis cases in the Baltic States. *PLoS One* 15:e0235841. 10.1371/journal.pone.0235841 32645064 PMC7347188

[B37] Marotta F. Di Marcantonio L. Janowicz A. Pedonese F. Di Donato G. Ardelean A. (2020). Genotyping and antibiotic resistance traits in *Campylobacter jejuni* and *coli* from pigs and wild boars in Italy. *Front. Cell. Infect. Microbiol.* 10:592512. 10.3389/fcimb.2020.592512 33178635 PMC7593542

[B38] Meistere I. Íibilds J. Eglîte L. Alksne L. Avsejenko J. Cibrovska A. (2019). *Campylobacter* species prevalence, characterisation of antimicrobial resistance and analysis of whole-genome sequence of isolates from livestock and humans, Latvia, 2008 to 2016. *Eur. Surveill* 24:1800357. 10.2807/1560-7917.ES.2019.24.31.1800357 31387670 PMC6685098

[B39] Mossong J. Mughini-Gras L. Penny C. Devaux A. Olinger C. Losch S. (2016). Human campylobacteriosis in Luxembourg, 2010-2013: a case-control study combined with Multilocus Sequence Typing for source attribution and risk factor analysis. *Sci. Rep.* 6:20939. 10.1038/srep20939 26860258 PMC4748240

[B40] Mourkas E. Florez-Cuadrado D. Pascoe B. Calland J. K. Bayliss S. C. Mageiros L. (2019). Gene pool transmission of multidrug resistance among *Campylobacter* from livestock, sewage and human disease. *Environ. Microbiol.* 21 4597–4613. 10.1111/1462-2920.14760 31385413 PMC6916351

[B41] Mourkas E. Taylor A. J. Méric G. Bayliss S. C. Pascoe B. Mageiros L. (2020). Agricultural intensification and the evolution of host specialism in the enteric pathogen *Campylobacter jejuni*. *Proc. Natl. Acad. Sci. U.S.A.* 117 11018–11028. 10.1073/pnas.1917168117 32366649 PMC7245135

[B42] Nachamkin I. Ung H. Li M. (2002). Increasing fluoroquinolone resistance in *Campylobacter jejuni*, Pennsylvania, USA,1982–2001. *Emerg. Infect. Dis.* 8 1501–1503. 10.3201/eid0812.020115 12498672 PMC2738503

[B43] Newell D. G. Mughini-Gras L. Kalupahana R. S. Wagenaar J. A. (2017). “*Campylobacter* epidemiology - Sources and routes of transmission for human infection,” in *Campylobacter*: Features. Detection, and Prevention on Foodborne Disease, ed. Klein G. (Amsterdam: Elsevier), 85–110. 10.1016/B978-0-12-803623-5.00005-8

[B44] Nguyen T. N. Hotzel H. El-Adawy H. Tran H. T. Le M. T. Tomaso H. (2016). Genotyping and antibiotic resistance of thermophilic *Campylobacter* isolated from chicken and pig meat in Vietnam. *Gut Pathog.* 8:19. 10.1186/s13099-016-0100-x 27175218 PMC4863348

[B45] Painset A. Day M. Doumith M. Rigby J. Jenkins C. Grant K. (2020). Comparison of phenotypic and WGS-derived antimicrobial resistance profiles of *Campylobacter jejuni* and *Campylobacter coli* isolated from cases of diarrhoeal disease in England and Wales, 2015-16. *J. Antimicrob. Chemother.* 75 883–889. 10.1093/jac/dkz539 31943013

[B46] Pascoe B. Schiaffino F. Murray S. Méric G. Bayliss S. C. Hitchings M. D. (2020). Genomic epidemiology of *Campylobacter jejuni* associated with asymptomatic pediatric infection in the Peruvian Amazon. *PLoS Negl. Trop. Dis.* 14:e0008533. 10.1371/journal.pntd.0008533 32776937 PMC7440661

[B47] Pollett S. Rocha C. Zerpa R. Patiño L. Valencia A. Camiña M. (2012). *Campylobacter* antimicrobial resistance in Peru: a ten-year observational study. *BMC Infect. Dis.* 12:193. 10.1186/1471-2334-12-193 22898609 PMC3482591

[B48] Price L. B. Johnson E. Vailes R. Silbergeld E. (2005). Fluoroquinolone-resistant Campylobacter isolates from conventional and antibiotic-free chicken products. *Environ. Health Perspect.* 113 557–560. 10.1289/ehp.7647 15866763 PMC1257547

[B49] Ramonaite S. Tamuleviciene E. Alter T. Kasnauskyte N. Malakauskas M. (2017). MLST genotypes of *Campylobacter jejuni* isolated from broiler products, dairy cattle and human campylobacteriosis cases in Lithuania. *BMC Infect. Dis.* 17:430. 10.1186/s12879-017-2535-1 28619013 PMC5472909

[B50] R Core Team (2019). *R: A Language and Environment for Statistical Computing.* Vienna: R Foundation for Statistical Computing.

[B51] Revez J. Rossi M. Ellström P. de Haan C. Rautelin H. Hänninen M.-L. (2011). Finnish *Campylobacter jejuni* strains of multilocus sequence type ST-22 complex have two lineages with different characteristics. *PLoS One* 6:e26880. 10.1371/journal.pone.0026880 22039552 PMC3200363

[B52] Rivera-Mendoza D. Martínez-Flores I. Santamaría R. I Lozano L. Bustamante V. H. Pérez-Morales D. (2020). Genomic analysis reveals the genetic determinants associated with antibiotic resistance in the zoonotic pathogen *Campylobacter* spp. distributed Globally. *Front. Microbiol.* 11:513070. 10.3389/fmicb.2020.513070 33042043 PMC7518152

[B53] Rokney A. Valinsky L. Moran-Gilad J. Vranckx K. Agmon V. Weinberger M. (2018). Genomic epidemiology of *Campylobacter jejuni* transmission in Israel. *Front. Microbiol.* 9:2432. 10.3389/fmicb.2018.02432 30386311 PMC6198274

[B54] Scallan E. Hoekstra R. M. Angulo F. J. Tauxe R. V. Widdowson M. A. Roy S. L. (2011). Foodborne illness acquired in the United States – major pathogens. *Emerg. Infect. Dis.* 17 7–15. 10.3201/eid1701.P11101 21192848 PMC3375761

[B55] Seemann T. *mlst. Github.* Available online at: https://github.com/tseemann/mlst

[B56] Sheppard S. K. Cheng L. Méric G. De Haan C. P. A. Llarena A.-K. Marttinen P. (2014). Cryptic ecology among host generalist *Campylobacter jejuni* in domestic animals. *Mol. Ecol.* 23 2442–2451. 10.1111/mec.12742 24689900 PMC4237157

[B57] Silva J. Leite D. Fernandes M. Mena C. Gibbs P. A. Teixeira P. (2011). *Campylobacter* spp. as a foodborne pathogen: a review. *Front. Microbiol.* 2:200. 10.3389/fmicb.2011.00200 21991264 PMC3180643

[B58] phac-nml/staramr *Staramr.* Available online at: https://github.com/phac-nml/staramr

[B59] Thépault A. Méric G. Rivoal K. Pascoe B. Mageiros L. Touzain F. (2017). Genome-wide identification of host-segregating epidemiological markers for source attribution in *Campylobacter jejuni*. *Appl. Environ. Microbiol.* 83 e03085-16. 10.1128/AEM.03085-16 28115376 PMC5359498

[B60] Van Boeckel T. P. Brower C. Gilbert M. Grenfell B. T. Levin S. A. Robinson T. P. (2015). Global trends in antimicrobial use in food animals. *Proc. Natl. Acad. Sci. U.S.A.* 112 5649–5654. 10.1073/pnas.1503141112 25792457 PMC4426470

[B61] Wang Y. Dong Y. Deng F. Liu D. Yao H. Zhang Q. (2016). Species shift and multidrug resistance of *Campylobacter* from chicken and swine, China, 2008–14. *J. Antimicrob. Chemother.* 71 666–669. 10.1093/jac/dkv382 26568567

[B62] Wieczorek K. Osek J. (2013). Antimicrobial resistance mechanisms among *Campylobacter*. *Biomed. Res. Int.* 2013:340605. 10.1155/2013/340605 23865047 PMC3707206

[B63] WHO (2017). *WHO Publishes List of Bacteria for Which New Antibiotics are Urgently Needed.* Geneva: WHO Media Centre.

[B64] Wysok B. Wojtacka J. Hänninen M.-L. Kivistö R. (2020). Antimicrobial resistance and virulence-associated markers in *Campylobacter* strains from diarrheic and non-diarrheic humans in Poland. *Front. Microbiol.* 11:1799. 10.3389/fmicb.2020.01799 32849410 PMC7417443

[B65] Yahara K. Méric G. Taylor A. J. de Vries S. P. W. Murray S. Pascoe B. (2017). Genome-wide association of functional traits linked with *Campylobacter jejuni* survival from farm to fork. *Environ. Microbiol.* 19 361–380. 10.1111/1462-2920.13628 27883255

[B66] Zankari E. Hasman H. Cosentino S. Vestergaard M. Rasmussen S. Lund O. (2012). Identification of acquired antimicrobial resistance genes. *J. Antimicrob. Chemother.* 67 2640–2644. 10.1093/jac/dks261 22782487 PMC3468078

[B67] Zankari E. Allesøe R. Joensen K. G. Cavaco L. M. Lund O. Aarestrup F. (2017). PointFinder: a novel web tool for WGS-based detection of antimicrobial resistance associated with chromosomal point mutations in bacterial pathogens. *J. Antimicrob. Chemother.* 72 2764–2768. 10.1093/jac/dkx217 29091202 PMC5890747

[B68] Zhao S. Mukherjee S. Chen Y. Li C. Young S. Warren M. (2015). Novel gentamicin resistance genes in *Campylobacter* isolated from humans and retail meats in the USA. *J. Antimicrob. Chemother.* 70 1314–1321. 10.1093/jac/dkv001 25645207 PMC11350565

[B69] Zhao S. Tyson G. H. Chen Y. Li C. Mukherjee S. Young S. (2016). Whole-genome sequencing analysis accurately predicts antimicrobial resistance phenotypes in *Campylobacter* spp. *Appl. Environ. Microbiol.* 82 459–466. 10.1128/AEM.02873-15 26519386 PMC4711122

[B70] Zirnstein G. Helsel L. Li Y. Swaminathan B. Besser J. (2000). Characterization of gyrA mutations associated with fluoroquinolone resistance in *Campylobacter coli* by DNA sequence analysis and MAMA PCR. *FEMS Microbiol. Lett.* 190 1–7. 10.1111/j.1574-6968.2000.tb09253.x 10981681

