# Supplementary material for: Whole Resistome Analysis in Campylobacter jejuni and C. coli Genomes Available in Public Repositories
Source: Front Microbiol. 2021 Jul 5;12:662144. doi: 10.3389/fmicb.2021.662144 (PMC8287256; doi:10.3389/fmicb.2021.662144)
Supplement: Supplementary file 3 [file Table_3.docx]

**Table S3.** Sequence Types within the most prevalent *C. jejuni* and *C. coli* CCs, classified by their host specialization according to results of the current study. Those STs with less than 20 genomes with a known non-human source of isolation were not employed for the specialist/generalist host association analysis.
