# Supplementary material for: Whole Resistome Analysis in Campylobacter jejuni and C. coli Genomes Available in Public Repositories
Source: Front Microbiol. 2021 Jul 5;12:662144. doi: 10.3389/fmicb.2021.662144 (PMC8287256; doi:10.3389/fmicb.2021.662144)
Supplement: Supplementary file 4 [file Presentation_1.pdf]

Supplementary Material

1 Supplementary Figures and Tables

Table S1. Metadata information for 37,398 *C. jejuni* genomes analyzed. Collection year, Continent, Country, and Host columns were manually inspected and modified to have labelling uniformity and reduce redundant groups. MLST information was obatined by staramr pipeline, as indicated on Methodology.

Table S2. Metadata information for 11,920 *C. coli* genomes analyzed. Collection year, Continent, Country, and Host columns were manually inspected and modified to have labelling uniformity and reduce redundant groups. MLST information was obatined by staramr pipeline, as indicated on Methodology.

Figure S1. Geographical resistome differences. Boxplots for prevalence of main ARGs or SNPs within clusters of (A) *C. jejuni* genomes by continent and (B) country; and of (C) *C. coli* genomes by continent and (D) countries. Each dot represent the prevalence on the AR determinant on a continent or country within the corresponding cluster and species. Only p-values lower than 0.05 are represented (t.test).

Figure S2. Host specialization of CCs and STs. Boxplots for prevalence of chicken and cow as host source of isolation for (A) C. *jejuni* CCs and (B) *C. jejuni* STs; and for (C) chicken and pig as host for *C. coli* STs. Each dot represent the prevalence of the AR determinant on a CC or ST within the corresponding host specialist/generalist group. Only p-values lower than 0.05 are represented (t.test).

Figure S3. Source isolation resistome differences. Boxplots for prevalence of main ARGs or SNPs within clusters of (A) *C. jejuni* and (B) *C. coli* genomes by host source of isolation. Each dot represent the prevalence of the AR determinant on genomes from the same host source of isolation within the corresponding cluster and species. Only p-values lower than 0.05 are represented (t.test).

Figure S4. *C. jejuni* CCs resistome differences. Boxplots for prevalence of main ARGs or SNPs within *C. jejuni* CC clusters of (A) main antibiotic families and (B) main ARGs or SNPs. Each dot represent the prevalence on the AR determinant on a CC within the corresponding cluster and species. Only p-values lower than 0.05 are represented (t.test).

Figure S4. *C. jejuni* resistome changes among last 20 years. Boxplots of temporal changes in *C. jejuni* European genomes for (A) the prevalence of determinants associated with resistance to the main antibiotic families, (B) the main ARGs or SNPs in all European genomes, (C) genomes from human isolates, (D) genomes from chicken isolates; and main genes co-occurrence patterns on (E) genomes from human isolates and (F) genomes from chicken isolates. Each dot represent the prevalence of the AR determinant on genomes from the same year of isolation within the corresponding 5-year period cluster. Only p-values lower than 0.05 are represented (t.test).
