# Supplementary figures and images for: Whole Resistome Analysis in Campylobacter jejuni and C. coli Genomes Available in Public Repositories
Source: Front Microbiol. 2021 Jul 5;12:662144. doi: 10.3389/fmicb.2021.662144 (PMC8287256; doi:10.3389/fmicb.2021.662144)

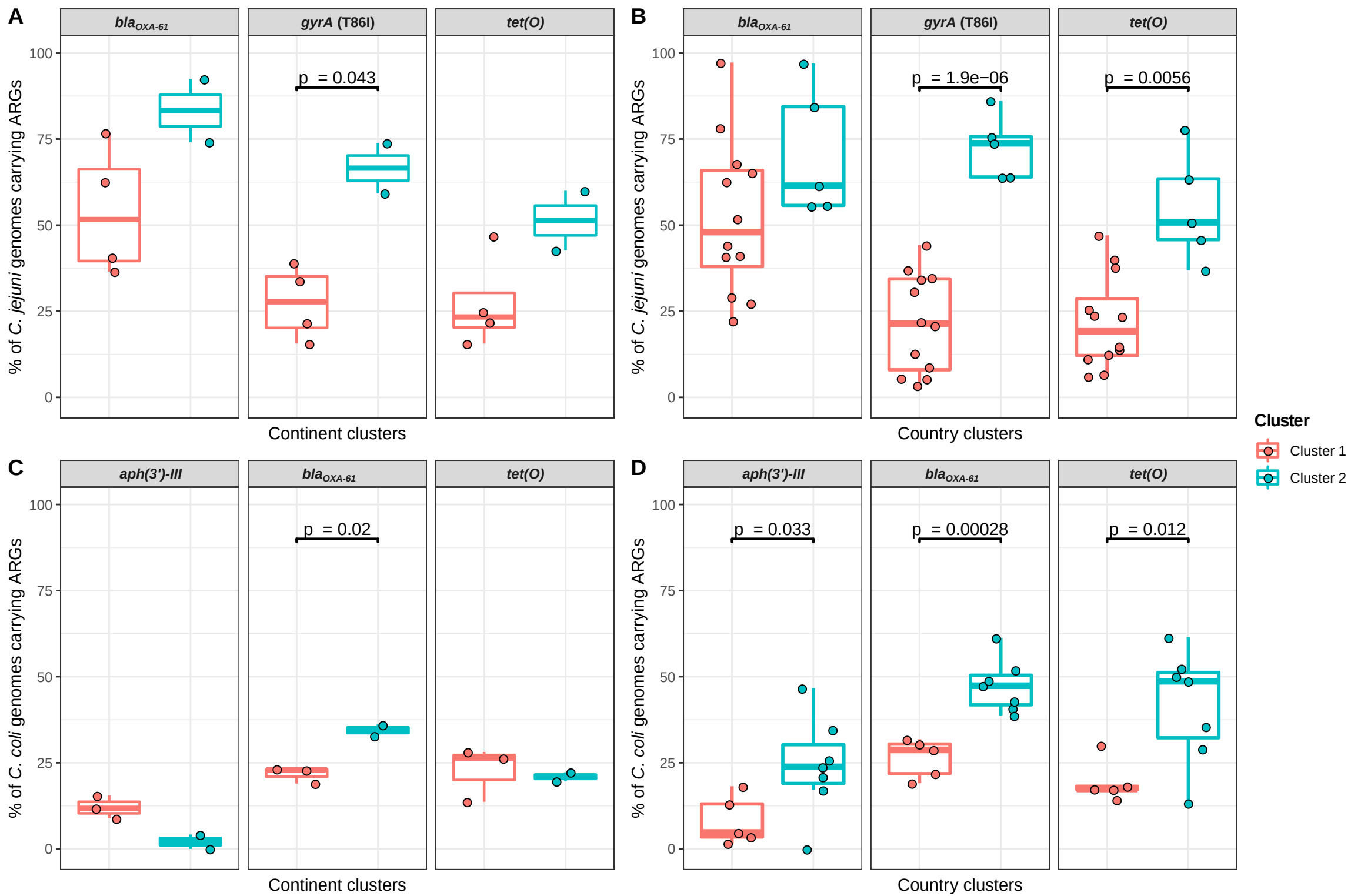

Supplement: Supplementary file 5 [file Data_Sheet_1.PDF]

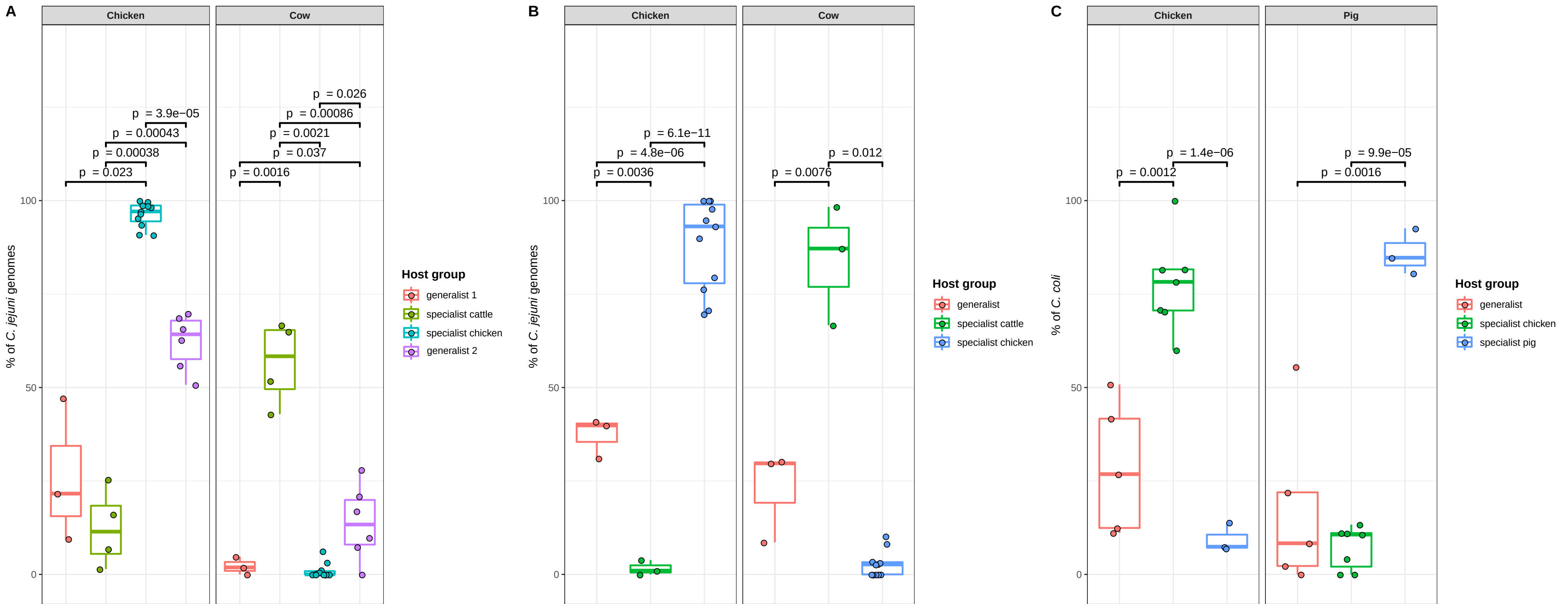

Supplement: Supplementary file 6 [file Data_Sheet_2.PDF]

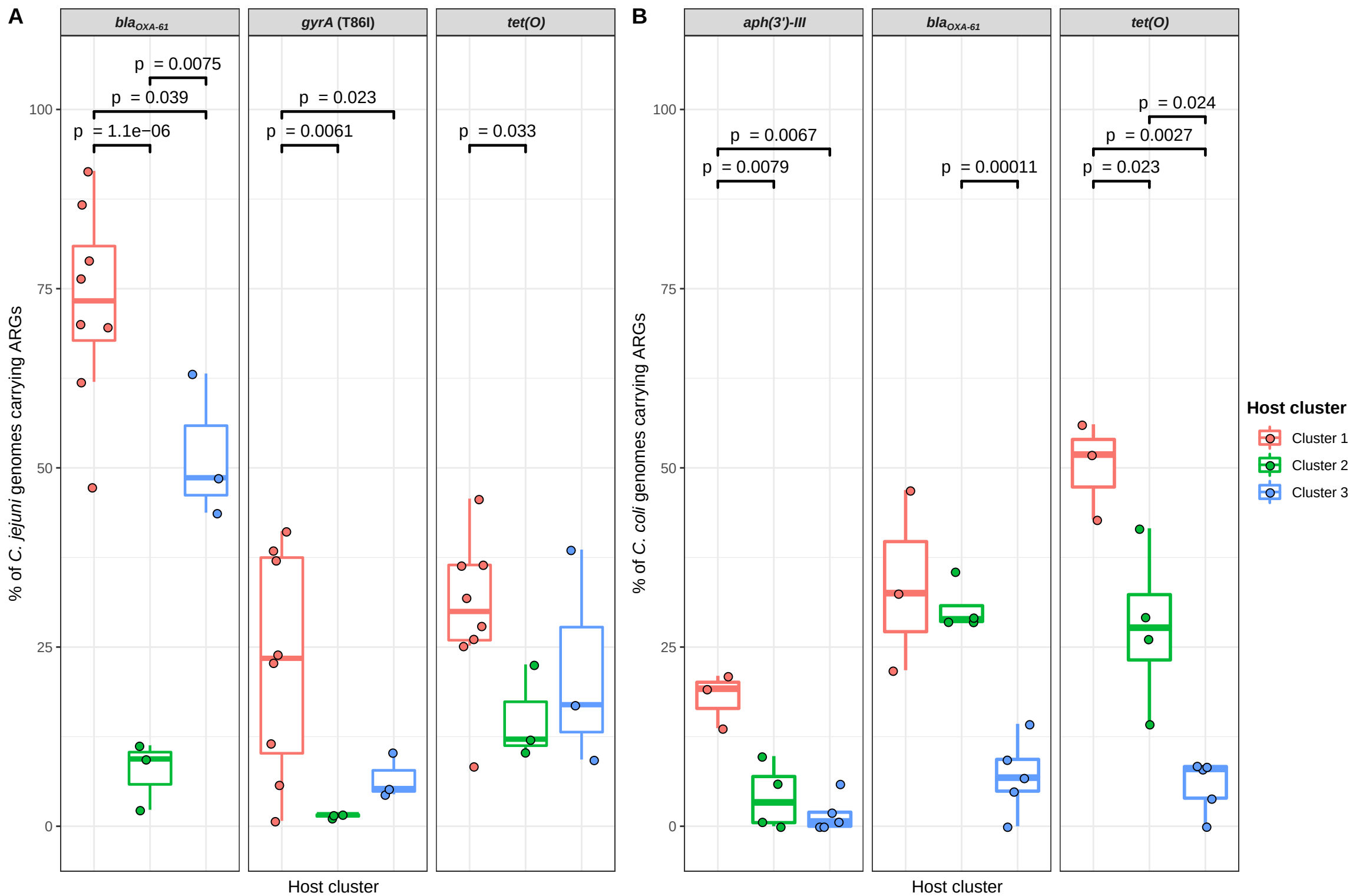

Supplement: Supplementary file 7 [file Data_Sheet_3.PDF]

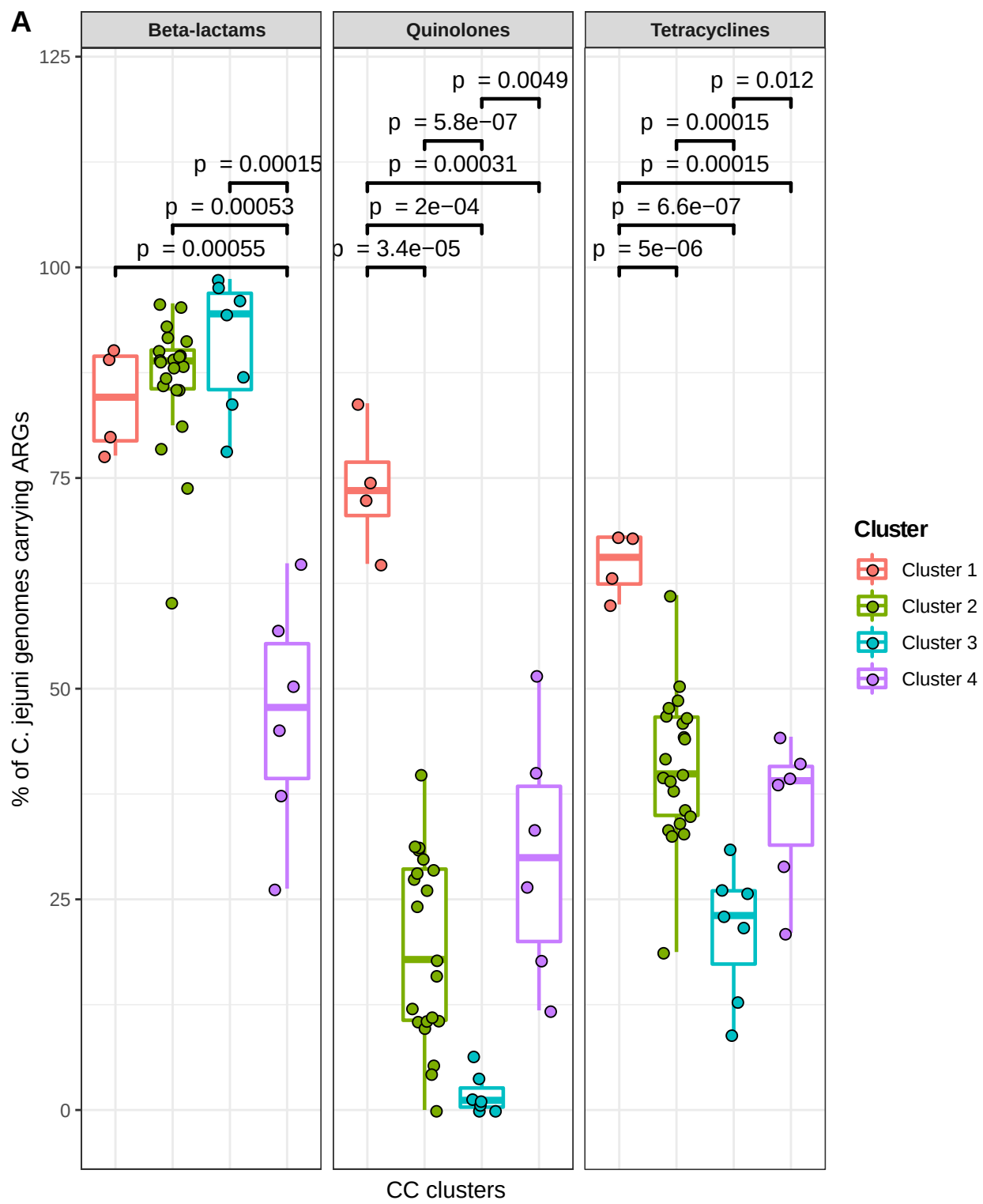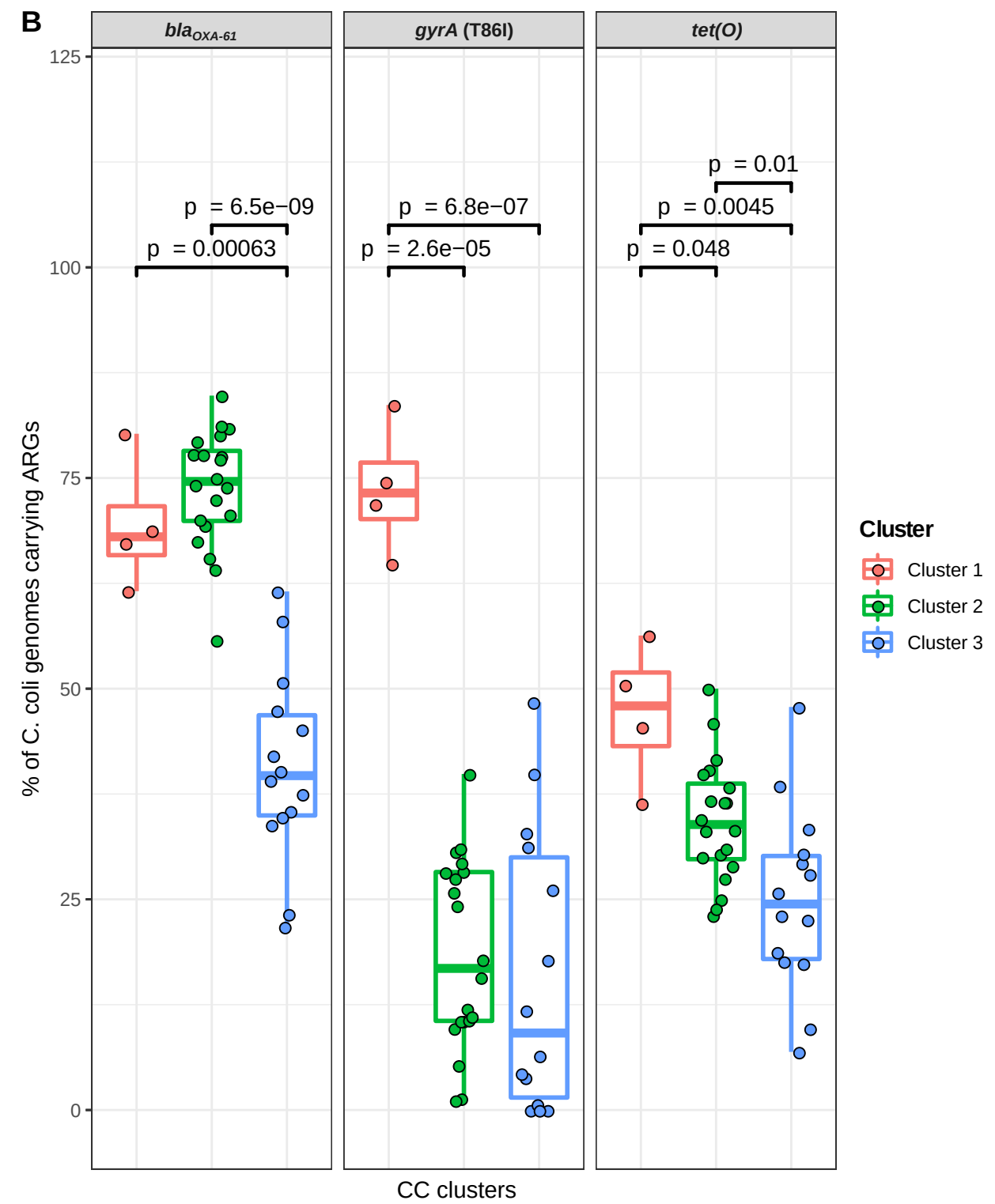

Supplement: Supplementary file 8 [file Data_Sheet_4.PDF]

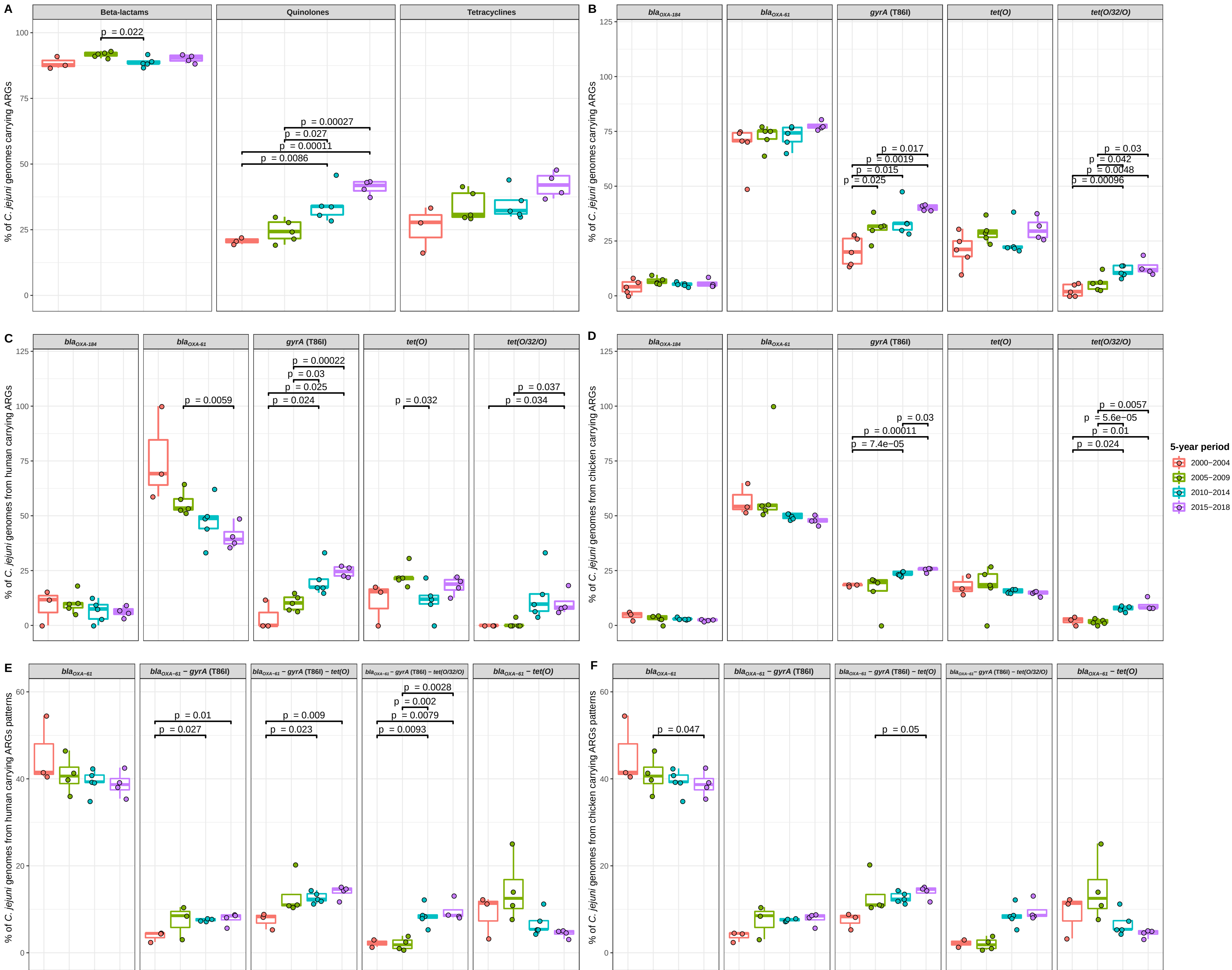

Supplement: Supplementary file 9 [file Data_Sheet_5.PDF]
